# ﻿Revision of the water scavenger beetle genus *Notionotus* Spangler, 1972 in the Neotropical Region (Coleoptera, Hydrophilidae, Enochrinae)

**DOI:** 10.3897/zookeys.1109.80775

**Published:** 2022-07-01

**Authors:** Liza M. González-Rodríguez, Andrew Edward Z. Short

**Affiliations:** 1 Department of Ecology & Evolutionary Biology, and Division of Entomology, Biodiversity Institute, University of Kansas, Lawrence, KS 66045, USA University of Kansas Lawrence United States of America

**Keywords:** aquatic insects, integrative taxonomy, new species, South America

## Abstract

The Neotropical species of the water scavenger beetle genus *Notionotus* Spangler, 1972 are revised using an integrative taxonomic approach combining morphology with DNA sequence data from two genes. Support exists for four putative species groups into which 18 species are placed, including twelve that are described here as new: *N.bicolor***sp. nov.** (Suriname), *N.bifidus***sp. nov.** (Venezuela), *N.brunbadius***sp. nov.** (Brazil), *N.garciae***sp. nov.** (Brazil), *N.giraldoi***sp. nov.** (Brazil), *N.insignitus***sp. nov.** (Venezuela), *N.juma***sp. nov.** (Brazil), *N.parvus***sp. nov.** (Suriname), *N.patamona***sp. nov.** (Guyana), *N.peruensis***sp. nov.** (Peru), *N.retusus***sp. nov.** (Guyana), and *N.vatius***sp. nov.** (Brazil). Four new synonymies are created: *N.shorti* Queney **syn. nov.** is found to be conspecific with *N.dilucidus* Queney; *N.edibethae* García **syn. nov.**, *N.nucleus* Perkins **syn. nov.**, and *N.perijanus* García **syn. nov.** are found to be conspecific with *N.tricarinatus* Perkins. New records are provided for all previously described species except *N.mexicanus* Perkins. Within the Neotropical region, the range of the genus is greatly expanded and now known from as far south as Bolivia and the Brazilian state of Mato Grosso do Sul. While a few species are found in hygropetric habitats, most are associated with the margins of forested streams. Genitalia and habitus images are provided for nearly all species, as well as a key to the four species groups.

## Abstract (es)

Las especies de escarabajos acuáticos detritívoros neotropicales del género *Notionotus* Spangler, 1972 son revisadas usando taxonomía integrativa, combinando morfología con datos de secuencias de ADN para dos genes. Se encontró soporte para cuatro grupos de especies conformados por 18 especies, incluyendo 12 que son aquí descritas como nuevas: *N.bicolor***sp. nov.** (Suriname), *N.bifidus***sp. nov.** (Venezuela), *N.brunbadius***sp. nov.** (Brazil), *N.garciae***sp. nov.** (Brazil), *N.giraldoi***sp. nov.** (Brazil), *N.insignitus***sp. nov**. (Venezuela), *N.juma***sp. nov.** (Brazil), *N.parvus***sp. nov.** (Suriname), *N.patamona***sp. nov.** (Guyana), *N.peruensis***sp. nov.** (Peru), *N.retusus***sp. nov.** (Guyana), *N.vatius***sp. nov.** (Brazil). Se sinonimizan cuatro especies: *N.shorti* Queney **syn. nov.** se considera conespecífico con *N.dilucidus* Queney; *N.edibethae* García **syn. nov.**, *N.nucleus* Perkins **syn. nov.**, y *N.perijanus* García **syn. nov.** son conespecíficos con *N.tricarinatus* Perkins. Nuevos registros son provistos para todas las especies previamente descritas excepto para *N.mexicanus* Perkins. Hasta el momento, en la región neotropical, el rango de distribución del género es ampliamente expandido, se conoce desde el Sur de Bolivia hasta el estado brasileño de Mato Grosso do Sul. Si bien algunas especies son encontradas en hábitats higropétricos, la mayoría de ellas están asociadas a las orillas de arroyos boscosos. Se proveen imágenes del hábito dorsal y genitalia para la mayoría de las especies, al igual que la clave para los cuatro grupos de especies.

## ﻿Introduction

The genus *Notionotus* Spangler, 1972 is a group of small to minute water scavenger beetles that occur in streams and seepages in the tropics of the New World and Southeast Asia. The original concept of *Notionotus* was based on two new species from the Venezuelan Andes (Spangler 1972). In the Neotropics, its range was soon expanded north to Mexico, with three species described by Perkins (1979) from Guatemala, Mexico and Panama, and subsequent additional species from Venezuela (García 2000). Most recently, three species were described from French Guiana and Guyana (Queney 2010) with additional new records being provided in a series of biotic surveys in Suriname and Guyana (Short and Kadosoe 2011; Short 2013; Short et al. 2017, 2018). *Notionotus* has also been reported from the Old World tropics, with seven species described from southeast India and Southeast Asia (Hebauer 2001, 2003) and southern China (Jia and Short 2011). Currently there are 17 described species, including ten from the Neotropics and seven from tropical Asia.

During the last two decades, extensive fieldwork for aquatic beetles in the Neotropics has resulted in the collection of many new specimens of *Notionotus*, including some that represent new species. We here use an integrative approach, combining adult morphological data with DNA sequences from two gene loci, to revise the Neotropical species of the genus. This revision provides a taxonomic foundation not only for future descriptive work on the genus, but also for evolutionary studies on the diversity and biogeography of the Neotropical region.

## ﻿Materials and methods

### ﻿Molecular methods

We amplified and sequenced the mitochondrial gene COI and the nuclear ribosomal gene 28S for 56 specimens of *Notionotus*, including 55 specimens from the Neotropical region and one unidentified specimen from India which we used as an outgroup to root the tree. We sampled specimens from most localities for which we had appropriate material, including a broad geographic sampling of *N.tricarinatus* and *N.dilucidus* as these two species had large ranges with some observed variation in the aedeagus. All specimens were preserved as frozen tissue samples since collection with the exception of the specimen from Costa Rica (SLE2381) which was pinned. Molecular extraction and sequencing methods follow those of Kohlenberg and Short (2017). Resulting DNA sequences were assembled and edited in Geneious R 8.0.5 (Biomatters, http://www.geneious.com/). All sequences are deposited in GenBank (see Table 1 for accession numbers). We used IQ-TREE 1.4.4 (Nguyen et al. 2015) to infer phylogenetic relationships. Each gene was placed in its own partition. The optimal models of substitution for each partition were selected using the Auto function in IQ-TREE 1.4.4. In order to assess nodal support, we performed 1000 ultrafast bootstrap replicates (Minh et al. 2013).

**Table 1.** List of DNA voucher specimens and GenBank accession numbers used in this study. “N/A” indicates the gene fragment was not successfully amplified or sequenced.

Taxon	Extraction	Locality	COI Accession	28S Accession
*N.bicolor*	SLE1810	Suriname: Sipaliwini: Kabalebo Nature Resort	ON239446	ON243733
*N.bicolor*	SLE2120	Suriname: Sipaliwini: Kabalebo Nature Resort	ON239447	N/A
*N.bifidus*	SLE1113	Venezuela: Amazonas: Tobogán de la Selva	ON239437	ON243725
*N.bifidus*	SLE2369	Venezuela: Amazonas: Tobogán de la Selva	ON239438	ON243726
*N.brunbadius*	SLE1553	Brazil: Amazonas: Ducke Reserve	ON239441	ON243731
*N.brunbadius*	SLE2102	Brazil: Amazonas: Ducke Reserve	ON239442	ON243732
*N.dilucidus*	SLE0505	Suriname: Brokopondo: Brownsberg Nature Park	ON239422	N/A
*N.dilucidus*	SLE0506	Suriname: Brokopondo: Brownsberg Nature Park	ON239420	ON243688
*N.dilucidus*	SLE1799	Suriname: Sipaliwini: Kabalebo Nature Resort	ON239418	ON243695
*N.dilucidus*	SLE1811	Suriname: Sipaliwini: Kabalebo Nature Resort	ON239419	ON243696
*N.dilucidus*	SLE2107	Guyana: Region IX: Kusad Mountain	ON239411	ON243702
*N.dilucidus*	SLE2108	Guyana: Region VI: Upper Berbice	ON239417	ON243692
*N.dilucidus*	SLE2113	French Guiana: Crique Eau Chire	ON239406	ON243693
*N.dilucidus*	SLE2114	Suriname: Sipaliwini: Sipaliwini Savanna Reserve	ON239413	ON243703
*N.dilucidus*	SLE2121	Suriname: Brokopondo: Brownsberg Nature Park	ON239421	ON243694
*N.dilucidus*	SLE2330	Brazil: Roraima: ca. 13 km NE of Caroebe	N/A	ON243704
*N.dilucidus*	SLE2335	Guyana: Region 9: Kusad Mountain	ON239412	ON243705
*N.dilucidus*	SLE2339	French Guiana: St. Laurent du Maroni (ca. 15 km SW)	ON239415	ON243690
*N.dilucidus*	SLE2366	Venezuela: Amazonas: Tobogán de la Selva	ON239404	ON243687
*N.dilucidus*	SLE2368	Guyana: Region 8: 7 km NW Chenapau	ON239409	ON243699
*N.dilucidus*	SLE2374	Brazil: Roraima: Rio Cocal, near Tepequem	ON239408	ON243698
*N.dilucidus*	SLE2375	Suriname: Sipaliwini: Wehepai	ON239416	ON243706
*N.dilucidus*	SLE2377	Brazil: Roraima: Serra do Tepequém	ON239410	ON243700
*N.dilucidus*	SLE2378	Venezuela: Bolívar: Piedra de la Virgen	ON239405	ON243701
*N.dilucidus*	SLE2380	Guyana: Region IX: Parabara	ON239414	ON243707
*N.dilucidus*	SLE2383	French Guiana: Carbet ONF Grillon	ON239407	ON243697
*N.dilucidus*	SLE2389	Suriname: Sipaliwini: Raleighvallen	ON239423	ON243689
*N.dilucidus*	SLE2394	Suriname: Sipaliwini: Raleighvallen	N/A	ON243691
*N.garciae*	SLE1900	Brazil: Amazonas: Pres. Fig. (ca. 57 km E)	ON239440	ON243730
*N.giraldoi*	SLE2088	Brazil: Rondonia: Vale do Cachoeiras	ON239428	ON243718
*N.giraldoi*	SLE2332	Brazil: Rondonia: Ji-Parana (27 km SW)	ON239427	ON243719
*N.giraldoi*	SLE2334	Brazil: Rondonia: Ji-Parana (27 km SW)	ON239429	ON243720
*N.insignitus*	SLE1115	Venezuela: Bolívar: La Escalera	ON239443	ON243734
*N.juma*	SLE1269	Brazil: Amazonas: Novo Airão	ON239435	ON243727
*N.juma*	SLE2100	Brazil: Amazonas: Ducke Reserve	ON239436	ON243728
*N.liparus*	SLE2111	Venezuela: Aragua: Henri Pittier National Park	ON239403	ON243714
*N.liparus*	SLE2123	Venezuela: Barinas: Barinitas (ca. 13 km NW)	ON239401	ON243715
*N.liparus*	SLE2124	Venezuela: Mérida: Santo Domingo (ca. 2 km SE)	ON239400	ON243716
*N.liparus*	MSC1820	Venezuela: Aragua: Henri Pittier National Park	ON239402	KC992598
*N.lohezi*	SLE2337	French Guiana: Savane Roche Virginie	ON239444	ON243737
*N.lohezi*	SLE2387	French Guiana: Savane Roche Virginie	ON239445	ON243738
*N.parvus*	SLE2388	Suriname: Sipaliwini: Grensgeberte Moutains	ON239434	ON243729
*N.retusus*	SLE2110	Guyana: Region 8: ca. 7 km NW Chenapau	N/A	ON243735
*N.retusus*	SLE2372	Guyana: Region 8: Ayanganna Airstrip	ON239439	ON243736
*N.tricarinatus*	SLE1112	Venezuela: Zulia: Tukuko	ON239425	ON243710
*N.tricarinatus*	SLE2371	Venezuela: Zulia: Tukuko	ON239426	ON243711
*N.tricarinatus*	SLE2381	Venezuela: Aragua: Henri Pittier National Park	ON239424	ON243712
*N.tricarinatus*	SLE2391	Venezuela: Portuguesa: Biscucuy	N/A	ON243713
*N.tricarinatus*	SLE2392	Venezuela: Portuguesa: Biscucuy	N/A	ON243708
*N.tricarinatus*	SLE2397	Costa Rica: Cartago: Tapanti National Park	N/A	ON243709
*N.vatius*	SLE2104	Brazil: Bahia: Cachoeira Domingos Lopes	ON239430	ON243722
*N.vatius*	SLE2324	Brazil: Mato Grosso do Sul: Aquidauana (ca. 27 km S)	ON239432	ON243724
*N.vatius*	SLE2327	Brazil: Mato Grosso do Sul: Aquidauana (ca. 15 km E)	ON239433	ON243723
*N.vatius*	SLE2385	Brazil: Bahia: Livramento de Nossa Senhora	ON239431	ON243721
*N.* sp.	SLE0092	India: Tamil Nadu: Bodi Hills, Western Ghats	ON239399	KC992599
*N.* sp.	SLE2140	Peru: Cusco: 1 km N. Quince Mil	ON239398	ON243717

### ﻿Morphological methods

We examined more than 900 specimens for this work, including the holotypes of most previously described Neotropical species. The specimens were examined using Olympus SZ61 and SZX7 microscopes and preparation of the specimens and dissection of the genitalia were carried out following the methodology of Girón and Short (2017). There was a modification in the time of exposure to heat of the genitalia during the clearing process depending on the level of sclerotization of the structure, which we reduced the time from 60 min to 30–40 min. Morphological terminology mainly followed Hansen (1991) and terminology adapted from Lawrence and Ślipiński (2013) regarding the use of meso- and metaventrite. Cleared genitalia photos were taken with Olympus BX51 microscope to 400× magnification (except for *N.rosalesi* genitalia to 100×), ~ 7–15 photos were taken per genitalia and stacked using CombineZP software. We generated the species distribution maps using the software SimpleMappr (Shorthouse 2010). The data on the holotype labels are provided verbatim and cited in quotation marks.

### ﻿Depositories of examined material

**CBDG**Center for Biological Diversity, University of Guyana, Georgetown (G. Maharaj);

**INPA**Instituto Nacional de Pesquisas da Amazônia, Manaus, Brazil (M. Oliveira);

**NMW**"Naturhistorisches Museum, Vienna, Austria (M. Jäch);

**MALUZ**Museo de Artrópodos de la Universidad del Zulia, Maracaibo, Venezuela (J. Camacho, M. García);

**MNHN**Muséum national d’Histoire naturelle, Paris, France;

**MIZA**Museo del Instituto de Zoología Agrícola, Maracay, Venezuela (L. Joly);

**NZCS**National Zoological Collection of Suriname, Paramaribo (P. Ouboter, V. Kadosoe);

**SCC** Private collection of Simon Clavier, Kourou, French Guiana;

**SEMC**Snow Entomological Collection, University of Kansas, Lawrence, KS (A. Short);

**UMSP**University of Minnesota Insect Collection, St. Paul, MN (R. Holzenthal, R.Thomson);

**USNM**U.S. National Museum of Natural History, Smithsonian Institution, Washington, DC (C. Micheli).

## ﻿Results

**Figure 1.**
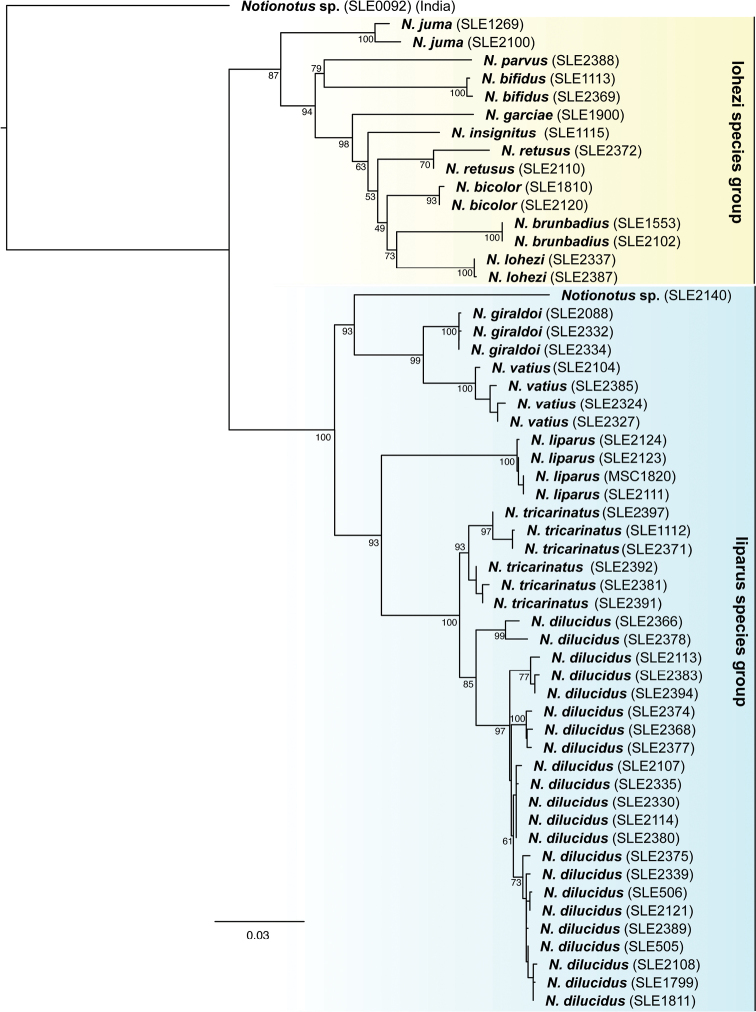
Maximum likelihood phylogeny of *Notionotus* spp. inferred from COI and 28S sequence data. Extraction numbers are given next to each terminal name (see Table 1).

From our integrated approach of combining morphological data with DNA sequence data from two genes, we found support for 18 species in the Neotropical region, including twelve new species. At the same time, we found that two of the most widespread species have been described multiple times, resulting in four new synonymies. For the 14 species that we had molecular data, the maximum likelihood analysis (Fig. 1) recovered two well-supported species groups. We found morphological characters that can be used to diagnose these two clades, which we here define as the *lohezi* and *liparus* species groups. In addition, two species that were not included in our molecular analysis are assigned to their own monotypic species groups as they present unique character combinations that were not consistent with either of the other species groups.

Among the 14 species for which we had molecular data, the minimum uncorrected pairwise distances in COI between any two species was 5.0% (between *N.giraldoi* and *N.vatius*) with the exception of *N.dilucidus*, in which the two specimens from Venezuela (SLE2366 and SLE2378) were 6% divergent from the remaining populations further to the east (Guyana, Brazil, Suriname, French Guiana). We sequenced one specimen from Peru that is known only from a single female, and we were unable to associate it with confidence to any of the described species for which we did not have DNA; however, due to the importance of the male genitalia in identifications, we refrain from describing this species here.

### ﻿List of species

#### *Notionotusliparus* species group

1. *Notionotusdilucidus* Queney, 2010: Brazil (Roraima), French Guiana, Guyana, Suriname, Venezuela

*Notionotusshorti* Queney, 2010, syn. nov.

1. *Notionotusgiraldoi* sp. nov.: Brazil (Rondonia)
2. *Notionotusliparus* Spangler, 1972: Venezuela
3. *Notionotusmexicanus* Perkins, 1979: Mexico
4. *Notionotustricarinatus* Perkins, 1979: Costa Rica, Guatemala, Panama, Venezuela

*Notionotusedibethae* García, 2000, syn. nov.

*Notionotusnucleus* Perkins, 1979, syn. nov.

*Notionotusperijanus* García, 2000, syn. nov.

1. *Notionotusvatius* sp. nov. :Brazil (Bahia, Mato Grosso do Sul)

#### *Notionotuslohezi* species group

1. *Notionotusbicolor* sp. nov.: Suriname
2. *Notionotusbifidus* sp. nov.: Venezuela
3. *Notionotusbrunbadius* sp. nov.: Brazil (Amazonas)
4. *Notionotusgarciae* sp. nov.: Brazil (Amazonas)
5. *Notionotusinsignitus* sp. nov.: Venezuela
6. *Notionotusjuma* sp. nov.: Brazil (Amazonas)
7. *Notionotuslohezi* Queney, 2010: Guyana
8. *Notionotusparvus* sp. nov.: Suriname
9. *Notionotuspatamona* sp. nov.: Guyana
10. *Notionotusretusus* sp. nov.: Guyana

#### *Notionotusperuensis* species group

1. *Notionotusperuensis* sp. nov.: Peru

#### *Notionotusrosalesi* species group

1. *Notionotusrosalesi* Spangler, 1972: Trinidad, Venezuela

### ﻿Taxonomy

#### ﻿Genus Notionotus

Taxon classificationAnimaliaColeopteraHydrophilidae

Spangler, 1972

90490B35-7B28-5A32-B923-C50F04517D50

Notionotus Spangler, 1972: 139.

##### Type species.

*Notionotusrosalesi* Spangler, 1972: 141; by original designation.

##### Differential diagnosis for Neotropical species.

**Figure 2.**
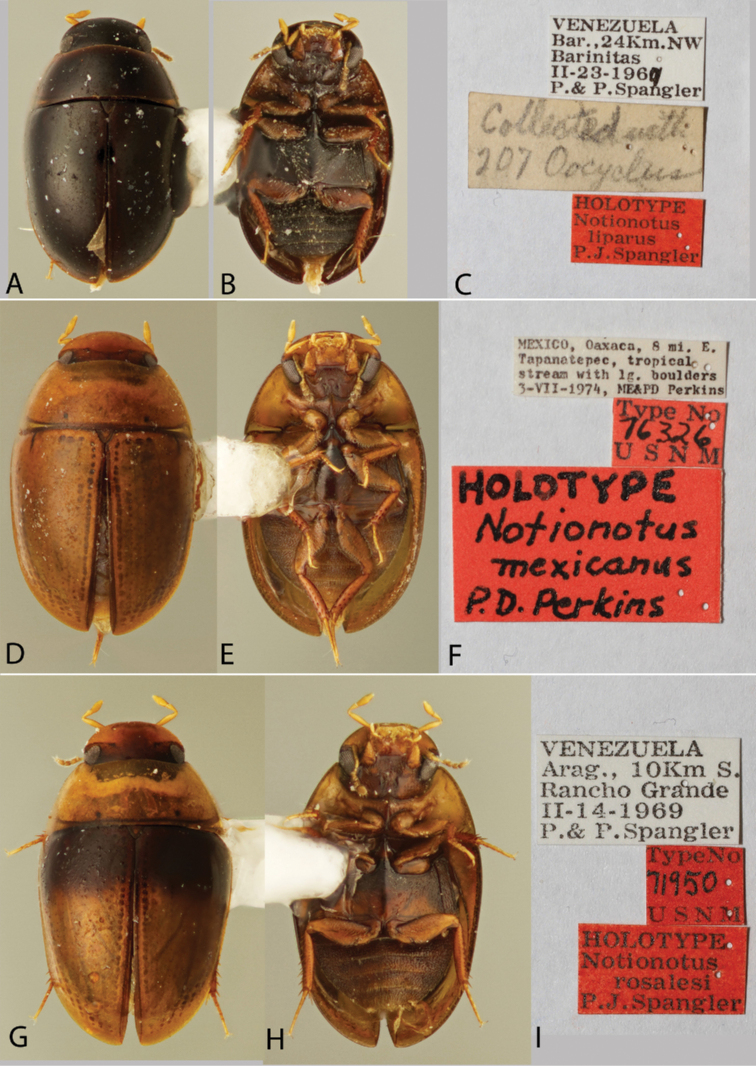
Habitus and labels of *Notionotus* spp.: *N.liparus* (holotype): **A** dorsal view **B** ventral view **C** labels; *N.mexicanus* (holotype): **D** dorsal view **E** ventral view **F** labels; *N.rosalesi* (holotype): **G** dorsal view **H** ventral view **I** labels.

**Figure 3.**
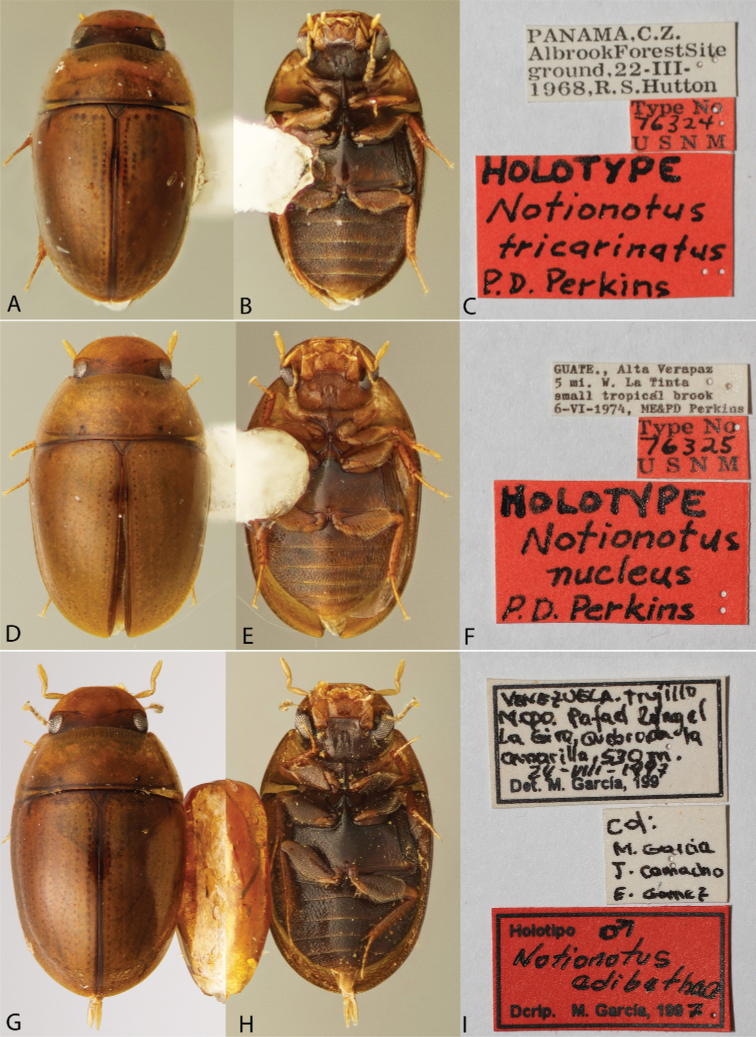
Habitus and labels of *Notionotus* spp.: *N.tricarinatus* (holotype): **A** dorsal view **B** ventral view **C** labels; *N.nucleus* (holotype): **D** dorsal view **E** ventral view **F** labels; *N.edibethae* (holotype): **G** dorsal view **H** ventral view **I** labels.

**Figure 4.**
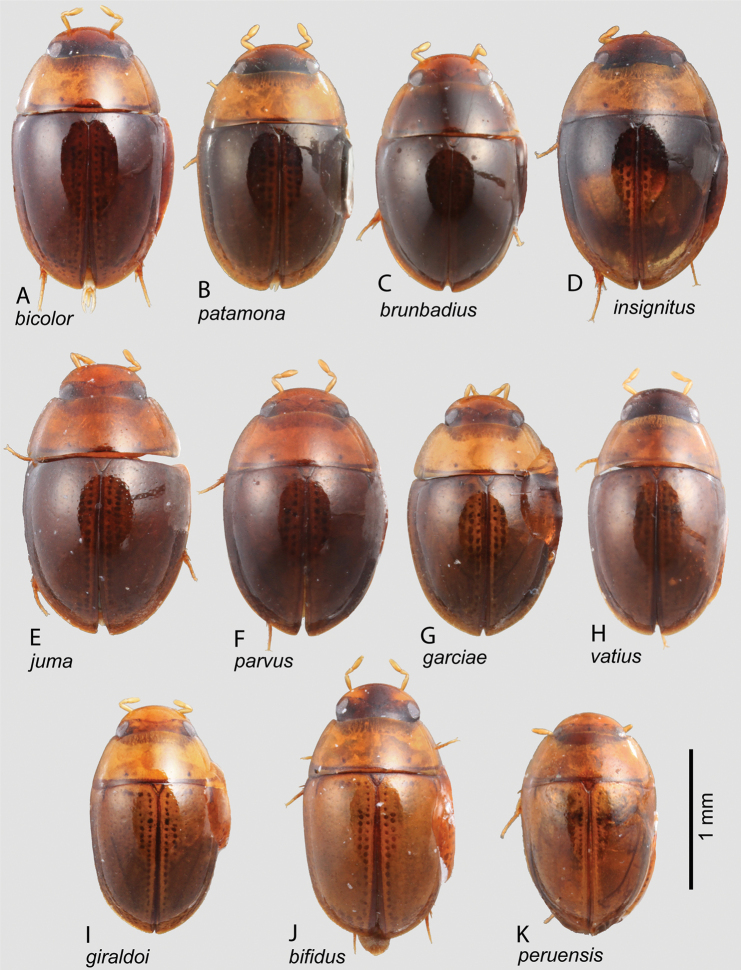
Habitus of *Notionotus* spp.: **A***N.bicolor* (paratype) **B***N.patamona* (holotype) **C***N.brunbadius* (holotype) **D***N.insignitus* (paratype) **E***N.juma* (paratype) **F***N.parvus* (holotype) **G***N.garciae* (paratype) **H***N.vatius* (holotype) **I***N.giraldoi* (paratype) **J***N.bifidus* (paratype) **K***N.peruensis* (holotype).

Small to very small beetles, total body length 1.5–2.0 mm. Color yellow, reddish brown, dark and pale brown to black. Body shape oval in dorsal view; moderately convex to convex in lateral view. Antennae with eight antennomeres. Maxillary palps short, nearly half the width of the head, second segment bending outwards, apical segment ~ 2 × as long as the penultimate segment (Fig. 4J; e.g., *N.bifidus* sp. nov.). Eyes reniform in dorsal view. Clypeus and labrum shallowly emarginate anteromedially, lateral margins of the labrum bearing setae. Head, pronotum and elytra with ground and systematic punctures; systematic punctures of the head very sparse. The elytral ground punctation is more evident in some species (Fig. 2A; e.g., *N.liparus*) than in others (Fig. 4G; e.g., *N.garciae* sp. nov.); systematic punctures extremely reduce and sparse detectable for short seta, forming very sparse rows (Fig. 4B; e.g., *N.patamona* sp. nov.); elytra without sutural stria. Prosternum carinate medially, strongly raised, and projected anteromedially. Elevation of mesoventrite strongly raised forming an anteromedial carina consisting of one (Fig. 10A, B) or two longitudinal ridges and one transverse (Fig. 10C, D), extending between procoxae on the same plane as metaventrite. Metaventrite densely pubescent, slightly elevated, with elevation broad posteromedially and convex medially forming a glabrous patch drop-shaped; and two posterolateral glabrous patches in a half-circle shape. Pro- and mesofemora mostly covered with pubescence on basal three-quarters (Fig. 3B; e.g., *N.tricarinatus*); metafemora with pubescence, sometimes on basal three-quarters, or along basal three-quarters of the anterior margin with some setae on the posterior basal margin (Fig. 2H; e.g., *N.rosalesi*). Abdominal ventrites densely pubescent, with fifth ventrite bearing an apical emargination to shallowly truncate. Aedeagus trilobed, size and form variable.

##### Remarks.

As this revision only treats the New World species, we did not comprehensively examine the Old World species to generate a global genus description. Therefore, the diagnosis above should be considered for Neotropical species only. The Old World species of *Notionotus* are generally similar to the New World species, but some species do differ in significant characters: for example, *N.suturalis* Hebaeur, 2003 has a sutural stria and antennae with 9 antennomeres.

##### Remarks of diagnostic features of *Notionotus* Spangler, 1972.

***Body shape and coloration*.** The degree of convexity between species is variable; some are moderately convex, others weakly convex. The general dorsal coloration of the body among species ranges from yellow (e.g., *N.tricarinatus*, Fig. 3A) to nearly black (e.g., *N.liparus*, Fig. 2A), however color alone is usually not sufficient on its own to definitively identify most *Notionotus* species. This is due both to the fact that some species share the same coloration, as well as some species have a slight variation in dorsal coloration. Species with unique (so far as currently known) color patterns include *N.liparus* (entirely dark brown to black, Fig. 2A), *N.rosalesi* (tricolored, Fig. 2G) and *N.insignitus* sp. nov. (with pale spot on the elytral disc Fig. 4D). The coloration of the head in some species is uniform, but in others is bicolorous (with typically the frons being darker than the clypeus).

***Mesoventrite*.** In *Notionotus* the elevation of the mesoventrite is composed of two or three laminae: one transverse ridge and one longitudinal (Fig. 10A, B) or two transverse ridges and one longitudinal ridge (Fig. 10C, D), which generally converge and fuse medially; the shape of the longitudinal ridge shows high variation among species. In general, the transverse ridges are medially elevated and laterally concave. The apex of the transverse ridge can be nearly acute (Fig. 10B), or blunt (Fig. 10C), and lateral sides vary from very to slightly concave or straight. The longitudinal ridge varies, it can be completely sharp or sharp anteriorly and broadening posteriorly reaching the end of the elevation, but it can also be broad anteriorly and sharp posteriorly. The point where the two or three ridges merged can be rounded and obtuse or wide and blunt respectively.

***Elytral punctation*.** The density of the ground punctation is typically sparse, and the degree of impression is variable between species within the genus. In some species, the ground punctation is very weakly impressed and may almost appear absent and low magnification (Fig. 4H; e.g., *N.vatius* sp. nov.); in other cases, it is more coarse and moderately impressed (Fig. 4E; e.g., *N.juma* sp. nov.).

***Aedeagus*.** The shape of the aedeagus is the most important and often crucial feature to identify species of *Notionotus*. Most species in the Neotropics exhibit two different generalized aedeagal forms: in some species, the median lobe and basal piece have the same length, or the median lobe is slightly longer than the parameres (e.g., *N.tricarinatus*, Fig. 6A). However, some species present the median lobe and basal lobe that are shorter than the parameres (e.g., *N.bicolor* sp. nov. (Fig. 8A), *N.retusus* sp. nov. (Fig. 8E), *N.parvus* sp. nov. (Fig. 9E)). In terms of shape, some species present variation in the apex of the median lobe, this is usually rounded (e.g., *N.patamona* sp. nov., Fig. 8D), but it varies from acute (e.g., *N.liparus*, Fig. 7A), emarginated (e.g., *N.juma*, Fig. 8G), and bifurcated (e.g., *N.bifidus* sp. nov., Fig. 9D). Additionally, the width of the median lobe varies from very slender (e.g., *N.giraldoi*, Fig. 7D) to very wide (e.g., *N.bifidus*, Fig. 9D). Nevertheless, in most of the species, the median lobe is wide at the base and slightly narrowing towards the apex.

###### *Notionotusliparus* species group

##### Diagnosis.

The species of this group can be diagnosed by the following combination of characters: (1) the shape of the elevation mesoventrite, having one transverse ridge and one longitudinal ridge (Fig. 10A, B); (2) parameres nearly as long as the basal piece; the length of the median lobe and the length of the parameres is approximately subequal. (e.g., *Notionotusliparus*, Fig. 7A).

#### ﻿ Notionotus dilucidus

Taxon classificationAnimaliaColeopteraHydrophilidae

Queney, 2010

CAA1758E-780E-5BE7-BBD0-1A536AFE704F

**Figure 5.**
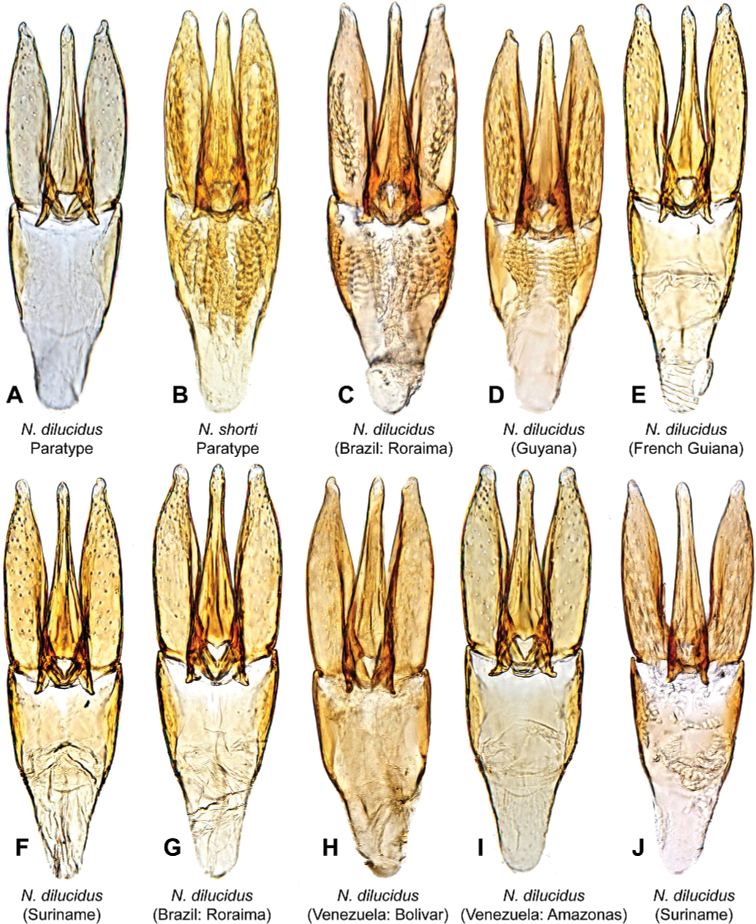
Aedeagi of *Notionotusdilucidus***A***N.dilucidus* (paratype) **B***N.shorti* (paratype) **C–J***N.dilucidus***C** specimen from Roraima, Brazil **D** specimen from Guyana **E** specimen from French Guiana **F** specimen from Suriname **G** specimen from Roraima, Brazil **H** specimen from Bolívar State, Venezuela **I** specimen from Amazonas State, Venezuela **J** specimen from Suriname.

Figs 5A–J , 10A , 15

Notionotus dilucidus Queney, 2010: 130.

##### Type material examined.

***Paratype* (male)**: “♂ ”, “*Notionotus*/*dilucidus* n. sp./ PARATYPE/ P. QUENEY descr. 2010”, “Guyane [= French Guiana]: Roura,/ Cacao, Chemin/ Molokoi, crique,/ 16-IX-2009/ leg. P. Queney” (SEMC). ***Paratypes* (8 exs.)**: same data as the dissected paratype (8 exs., SEMC).

#### ﻿ Notionotus shorti

Taxon classificationAnimaliaColeopteraHydrophilidae

Queney, 2010: 133. syn. nov.

5BD081AD-E949-546C-A160-3FEFBEAAACE9

##### Type material examined.

***Holotype* (male)**: “♂ ”, “*Notionotusshorti*/n. sp. HOLOTYPE/P. QUENEY descr.2010”, “Guyana: Mazaruni-Potaro/District, Takutu Mountains,/ stream debris berlesed, 18-/XII-1983, leg. P.J. Spangler,/ W.E. Steiner & M. Levine” (USNM). ***Paratypes* (8 exs.)**: same data as the holotype (8 exs., SEMC).

##### Additional material examined

**(362 exs.). Brazil: Roraima State**: Serra do Tepequém, 3°47.334'N, 61°42.570'W, 14.i.2018, leg. Short, Benetti, & Santana, small forested stream, BR18-0114-02A (1 ex., SEMC, DNA voucher SLE2377); Caroebe, Rio Caroebe, ca. 13 km NE of Caroebe, 00°54.786'N, 59°34.397'W, 150 m, 17.i.2018, leg. Short and Benetti, margins of sandy river, BR18-0117-04A (26 exs., INPA, SEMC, including DNA voucher SLE2330); Rio Jatapú nr. Usina de Jatapú, 00°50.939'N, 59°18.262'W, 145 m, 17.i.2018, leg. A. Short, marginal pools of river, BR18-0117-02A (1 ex., SEMC); Amajari, Rio Cocal, 3°44.135'N, 61°43.542'W, 237 m, 15.i.2018, leg. A. Short, clearwater creek, BR18-0115-01A (6 exs., SEMC, including DNA voucher SLE2374). **French Guiana**: St. Laurent du Maroni ca. 15 km SW, Crique des cascades, 5.34662, -54.10539, 17 m, 4.iii.2020; leg. Short and Neff, stream margin, FG20-0304-02A (1 ex., SEMC); same data except leg. Short, leaf packs on rock, FG20-0304-02B (2 exs., SEMC, including DNA voucher SLE2339); same data except small rock pools in granite FG20-0304-02C (1 ex., SEMC); Carbet ONF Grillon, Piste Bélizon, Crique Grillon, 4.28219, -52.45163, 65 m, 11.iii.2020; leg. Short, rock pools by waterfall, FG20-0311-01C (2 exs., SEMC, including DNA voucher SLE2383); Crique à l’Est above Crique Eau Chire, 3.66383, -53.22193, 156 m, 10.xi.2016, leg. D. Post (1 ex., SEMC, DNA voucher SLE2113). **Guyana: Region 6**: Upper Berbice, ca. 1 km, W basecamp, 4°09.143'N, 58°11.207'W, 170 m, 22.ix.2014, leg. Short, Salisbury, La Cruz, margins of creek, GY14-0921-03H (6 exs., SEMC); Upper Berbice, Basecamp 1, creek next to basecamp (upstream), 4.154817, -58.178616, 24.ix.2014, leg. Short, creek margins, GY14-0924-01A (1 ex., SEMC, DNA voucher SLE2108); Upper Berbice, Basecamp 1, 4°09.289'N, 58°10.717'W, 96 m, 22.ix.2014, leg. Short, Salisbury, La Cruz, GY14-0924-01A (2 ex., SEMC). **Region 8**: Upper Potaro Camp I (ca. 7 km NW Chenapau), 5°0.673'N, 59°38.358'W, 500 m, 14.iii.2014, leg. Short, Salisbury, La Cruz, clear water creek rapids, GY14-0314-01A (7 exs., SEMC); same data except 5°0.660'N, 59°38.283'W, 484 m, 11.iii.2014, leg. Short, Baca, Salisbury, La Cruz, clear water creek rapids, GY14-0311-04A (6 exs., SEMC, including DNA voucher SLE2368); same data except Potaro margin trail, 5°0.571'N, 59°38.202'W, 524 m, 11.iii.2014, leg. Short and Baca, leaf packs in rocky stream, GY14-0311-05A (1 ex., SEMC). **Region 9**: Kusad, Mts., Mokoro Creek, 2°48.531'N, 59°51.900'W, 170 m, 27.x.2013, leg. Short, Isaacs and Salisbury, main seepage area, GY13-1027-03B (2 exs., SEMC); same data except basecamp, leg. A. Short and Washington, on wet rocks, GY13-1024-03C (4 exs., SEMC); same data except 24.x.2013, leg. Salisbury, small rock pool with detritus, GY13-1024-03A (1 ex., SEMC); Kusad, Mts., basecamp area, 2°48.588'N, 59°51.931'W, 194 m, 23.x.2013, leg. A. Short, leaf packs in flow of creek, GY13-1023-02B (9 exs., CDBG, SEMC, including DNA voucher SLE2335); Kusad, Mts., Taraara Wao, 2°47.417'N, 59°53.986'W, 113 m, 28.x.2013, leg. Short, Isaacs and Salisbury, margin and isolated side pools, GY13-1028-01A (5 exs., SEMC, including DNA voucher SLE2107); N. Parabara, creek by basecamp (Bototo wau creek), 2°10.908'N, 59°20.306'W, leg. Short, 31.x.2013, stream margins, GY13-1031-01A (1 ex., SEMC, DNA voucher SLE2380). **Region Mazaruni-Potaro**: Takutu Mountains, 6°15'N, 59°5'W, 2–14.xii.1983, leg. P.D. Perkins, stream ex. leaf packs and twigs (8 exs., SEMC) **Suriname: Brokopondo District**: Brownsberg Nature Park, near Capaci House, 4°56.934'N, 55°10.825'W, 460 m, 17.iii.2017, leg. A. Short, small stream, SR17-0317-01A (1 ex., SEMC); Leo Val/lrene Val return loop trail, 4.95069'N, -55.18599'W, 470 m, 18.iii.2017, leg. Baca and Johnson, stream, SR17-0318-01B (1 ex., SEMC); Leo Val, 4°57'16.08"N, -55°11'26.82"W, 317 m, 19.iii.2017, leg. Short, leaf packs/detritus from behind waterfall, SR17-0319-01C (5 exs., SEMC); same data except SR17-0323-01C (6 exs., SEMC); same data except 23.iii.2017, leg. Baca, submerged woody debris, SR17-0323-01A (1 ex., SEMC); same data except side pools in creek, SR17-0323-01D (1 ex., SEMC); Witti Kreek, 4°55.674'N, 55°09.874'W, 84 m, 21.iii.2017, leg. Short and Baca, small side stream, SR17-0321-01D (2 exs., SEMC, including DNA voucher SLE2121); Brownsberg Nature Park, Mazaroni Val, 04°56.351'N, 55°12.108'W, 394 m, 5.viii.2012, leg. Short, Maier, Mcintosh, forested waterfall and stream, SR12-0805-01A (22 exs., NZCS, SEMC); Brownsberg Nature Park, 04°57.268'N, 55°11.447'W, 317 m, 4.viii.2012, leg. Short, Maier, Mcintosh, forested waterfall and stream, SR12-0804-02A (21 exs., SEMC); Brownsberg Nature Park, 04°56.871'N, 55°10.911'W, 462 m, 4.viii.2012, leg. Short, Maier, McIntosh, SR12-0804-01A (2 exs., SEMC including SLE505 and SLE506). **Sipaliwini District**: Kabalebo Nature Resort: Bwkw rapids, 4.40041°N, 57.24658°W, 90 m, 9–10.9iii.2019, leg. Short and class, large isolated muddy pool by river, SR19-0309-02A (1 ex., SEMC); Moi Moi creek, 4.42313°N, 57.19198°W, 104 m, 10–14.iii.2019, leg. Short and class, rock and detrital pools along creek, SR19-0310-01A (3 exs., SEMC); same data except leg. Short and Baca, small seeps, SR19-0310-01F (10 exs., SEMC); same data except leg. Short, margin of detrital pool in drying creekbed, SR19-0310-01G (5 exs., SEMC); same data except leg. Short, Baca and class, SR19-0310-01J (1 ex., SEMC); same data except leg. Baca, margin of stream pool with root mats, SR19-0310-01L (5 exs., SEMC); same data except leg. Short and class, SR19-0310-01M (10 exs., SEMC, including DNA voucher SLE1799); same data except Leg. S. Baca, upstream riparian habitats, SR19-0310-01N (2 exs., SEMC); Sand Crk, 4.38476°N, 57.24636°W, 72 m, 13–15.iii.2019, leg. Short and Baca, upper marginal pool along river, SR19-0313-01D (2 exs., SEMC); Charlie Falls, 4.38302°N, 57.21161°W, 174 m, 11.iii.2019, leg. Short and class, rock pools in creekbed, SR19-0311-01A (39 exs., NZCS, SEMC, including DNA voucher SLE1811); same data except leg. Short, seepage, SR19-0311-01B (7 exs., SEMC); Sipaliwini Savanna Nature Res. 4-Brothers Mts, 2°00.342'N, 55°58.149'W, 337 m, 31.iii.2017, leg. Short and Baca, clear water stream sandy with detritus, SR17-0331-01B (31 exs., SEMC, including DNA Voucher SLE2114); same data except leg. Baca, small rocky creek, SR17-0331-01A (1 ex., SEMC); Raleighvallen Nature Reserve, Fungu Island, 4°43.459'N, 56°12.658'W, 30 m, 14.iii.2016, leg. A. Short, isolated river margin pools, rocky bottom, SR16-0314-01E (1 ex., SEMC); Coppename Rvr-Voltzberg trail, 15.iii.2016, leg. A. Short, small sandy stream, SR16-0315-02A (1 ex., SEMC); Lolopaise Area, 4°42.48'N, 56°13.15908'W, 24 m, 18.iii.2016, leg. Short et al., intermittent stream margins, flotation, SR16-0318-01D (1 ex., SEMC); Brownsberg Nature Park, 04°56.871'N, 55°10.911'W, 462 m, 4.viii.2012, leg. Short, Maier, Mcintosh, forested stream with lots of detritus, SR12-0804-01A (8 exs., SEMC); Raleighfallen Nature Reserve, trail to Raleighfallen, 04°42.480'N, 56°13.159'W, 24 m, 27.vii.2012, leg. Short, Mcintosh and Kadosoe, creek margins, SR12-0727-03A (22 exs., SEMC, including DNA voucher SLE2394); same data except leg. C. Mcintosh, detrital pools near creek in forest, SR12-0727-03D (1 ex., SEMC); Raleighvallen Nature Reserve Voltzberg Station, 04°40.910'N, 56°11.138'W, 78 m, 9.vii.2012, leg. Short, Maier, McIntosh, and Kadosoe, stream margins, SR12-0729-02A (1 ex., SEMC, DNA voucher SLE2389); Raleighfallen Nature Reserve, Voltzberg trail, 04°40.910'N, 56°11.138'W, 78 m, 30.vii.2012, leg. C. Maier and V. Kadosoe, margin of stream, SR12-0730-01A (1 ex., SEMC); Raleighfallen Nature Reserve, Fungu island, 04°43.459'N, 56°12.658'W, 30 m, 1.viii.2012, leg. Short, Maier, Mcintosh and Kadosoe, small creek, SR12-0801-01B (2 exs., SEMC); Camp 2, on Sipaliwini District, CI-RAP Survey, 2°10.973'N, 56°47.235'W, 210 m, 29–30.viii.2010, leg. Short and Kadosoe, Inselberg, SR10-0829-01A (2 exs., SEMC); Camp 1, Upper Palumeu, CI-RAP Survey, 2.47700°N, 55.62941°W, 275 m, 11.iii.2012, leg. Short and Kadosoe, around waterfall, SR12-0311-03A (2 exs., SEMC); Camp 3, Wehepai, 2°21.776'N, 56°41.861'W, 237 m, 4–6.ix.2010, leg. Short and Kadosoe, sandy forest creek, SR10-0904-01A (36 exs., SEMC, including DNA voucher SLE2375). **Venezuela: Bolívar State**: Piedra de la Virgen, 6°5'14.1"N, 61°23'55.8"W, 400 m, 31.vii.2008, leg. A. Short M. García, L. Joly, small forest stream, AS-08-056 (7 exs., SEMC, including DNA voucher SLE2378). **Amazonas State**: Tobogán de la Selva, 5°23.207'N, 67°36.922'W, 125 m, 14.i.2009, leg. A. Short, clumps of wet leaves on rock, VZ09-0114-01D (6 exs., MIZA, SEMC, including DNA voucher SLE2366).

##### Differential diagnosis.

The general coloration of *Notionotusdilucidus* is similar to *N.giraldoi*, *N.mexicanus*, and *N.tricarinatus* and these species are very difficult to differentiate with external characters alone. However, the shape of the aedeagus in *N.dilucidus* is quite distinct: the outer and inner margins of the parameres are sinuate, the apex of the parameres presents an indentation in which the depth can vary from very deep, slightly deep, or almost not distinguishable and pointing outwards (Fig. 5A–J).

##### Description.

***Size and form***: Body length 1.6–1.8 mm. Body form elongate oval, moderately convex in lateral view (e.g., Fig. 2D). ***Color and punctation***: Dorsally yellow to pale brown, head mostly yellow and frons pale brown; pronotum paler than elytra, with two small black round spots along posterior margin (e.g., Fig. 2D). Ventrally brown; maxillary palps and antennae yellow (antennal club slightly darker), mouth parts and legs pale brown. Clypeus and labrum with dense, fine, and weakly impressed ground punctation (punctures separated by 1–2 × their width); pronotum and elytra with ground punctation fine, weakly impressed and sparser than on head (punctures separated by 3 × their width). ***Head***: Clypeus and labrum shallowly emarginate anteromedially, lateral margins of the labrum bearing setae. ***Thorax***: Prosternum carinate medially, strongly raised, pointing anteriorly and acute. Elevation of mesoventrite with one transversal ridge, elevated medially, lateral sides concave; longitudinal ridge sharp, the point where the two ridges merged rounded and obtuse (Fig. 10A); elevation flat in lateral view; mesoventrite with triangular shape in ventral view. Metaventrite convex in the median region, pubescent with narrow glabrous patch on the medial and posterolateral area, medial region patch drop-shaped; anterior margin extending to mesoventrite elevation. Metafemora densely covered with hydrofuge pubescence on basal three-quarters (e.g., Fig. 2E). ***Abdomen***: Abdominal ventrites very densely pubescent. Aedeagus (Fig. 5A) basal piece nearly the same length of a paramere. Base of the parameres slightly narrower than the base of the median lobe; outer margins sinuate, tapering almost reaching the apex, inner margins sinuate, apex of the parameres slightly bending outwards, rounded in the outer margin with an indentation in the inner margin (the depth of the indentation varies, Fig. 5A–J). Median lobe nearly as long as the parameres or a bit longer, broad at the base and gradually narrowing to the apex, apex of the median lobe rounded.

##### Variation.

There is significant variation in the apex of the parameres of the aedeagus. The paramere tip is weakly sclerotized, as evidenced by its very pale to white appearance in cleared specimens (e.g., Fig. 5A–J). This tip varies from nearly evenly rounded (e.g., Fig. 5D, G) to possessing some level of indentation on the inner margin (e.g., Fig. 5A, H). Although this variation seems in part to be real, it also seems to be partly caused by artificial distortions; for example, the weakly sclerotized apex may become indented or inflated depending on the state of the genitalia during preparation. This would explain why there is a large variation in form as well as why the tip appears to be exhibit subtly asymmetry between parameres (e.g., Fig. 5C, F).

##### Distribution.

This species is widely distributed throughout the Guiana Shield region, from Tobogán de la Selva in Venezuela east to the coast of French Guiana (Fig. 15). Reported from Suriname in Short and Kadosoe (2011) and Short (2013; as *N.shorti*) and from Guyana as *Notionotus* sp. B in Short et al. (2018).

##### Life history.

This species is the most common and abundant species of the genus in the Guiana Shield region and is frequently collected in leaf packs or along the margins of forested streams. They are typically found in streams that are lined with detritus and have rocky or sandy substrates. Although some specimens have been collected in seepage-like habitats or adjacent to seepages, this does not seem to be a primary or favored habitat for this species.

##### Remarks.

Queney (2010) described *N.dilucidus* based on specimens from several closely situated localities in French Guiana, and *N.shorti* based on a long series of specimens from a single collecting event in Guyana. The primary feature used to separate the two taxa was the apex of the parameters, which was given as “parameres apically shortly curved outwards” in *N.dilucidus* and “parameres apically almost straight” in *N.shorti*. We examined material from the type series of both species and confirmed there are notable differences in the paramere shape. If we only had access to the specimens that Queney (2010) examined and no other data, we also would have very likely concluded they were different species and described them as such. However, as we examined recently collected specimens from numerous other localities in the eastern Guiana Shield, we observed quite a bit of variation in the shape of the parameres that encompassed the forms present in *N.dilucidus* and *N.shorti*. In some cases, we noticed variation in this feature even among specimens from the same series. Additionally, the apex of the paramere even occasionally appears slightly asymmetrical in some dissections (e.g., Fig. 5F, J).

To help interpret these morphological observations, we sequenced 22 specimens that span the entire width of the Guiana Shield, including specimens from Venezuela, Brazil, Guyana, Suriname, and French Guiana. These specimens also represented a range of paramere form diversity. In general, we found little meaningful molecular divergence among these populations (Fig. 1) and these differences were not correlated to observed variations in the paramere apex. Therefore, we here consider *N.shorti* as a junior subjective synonym of *N.dilucidus*.

Among all sequenced specimens, the maximum pairwise divergence in the gene COI was 6.0%. Although this is on the higher end of intraspecific divergence observed in hydrophilids, it is seen in some taxa (see Short and Girón 2018; Smith and Short 2020). Additionally, given that its geographic range spans 1500 kilometers, it is not surprising to see such divergences. The two Venezuelan populations (from Tobogán de Selva in Amazonas State and the Escalera region of Bolívar State) were the most genetically distinct and sister to the more eastern populations. Indeed, with these samples removed, the maximum intraspecific divergence among remaining specimens drops to just 3.8%. However, we did not observe any significant morphological differences from these Venezuelan populations and therefore consider them to be part of a broader definition of *N.dilucidus*.

As both *N.dilucidus* and *N.shorti* were proposed in the same work (Queney 2010), we use our authority as first revisors (ICZN article 24.2.2) to give precedence to *N.dilucidus* as the valid name for this species.

#### ﻿ Notionotus giraldoi sp. nov.

Taxon classificationAnimaliaColeopteraHydrophilidae

DE5FE268-20BA-5CAA-B1C5-2315B54C204C

http://zoobank.org/05F6195D-EB81-47FB-B7FD-2256F49D877F

Figs 4I , 7D , 13A , 14

##### Type material.

***Holotype* (male)**: “BRAZIL: Rondonia: Novo [*sic*: Nova] Uniao/ -10.91764°, -62.377°, 359 m/ Vale do Cachoeiras; 10.vii.2018/ leg. Short; Margin of rocky/ stream; BR18-0710-02B” (INPA). ***Paratypes* (56 exs.): Brazil: Mato Grosso do Sul State**: Rio Bento Gomes (Pantanal), Campo Allegre I, 15°45'S, 56°33'W, 1993–1994, leg. E. Stuhr, spring-fed brook, (9 exs., NMW, SEMC). **Rondonia State**: Same data as holotype (13 exs., SEMC, including DNA Voucher SLE2334); same data except margin of rocky stream with waterfall (12 exs., SEMC); same data except small sandy-bottom stream margin BR18-0710-02A (20 exs., INPA, SEMC, including DNA voucher SLE2088); same data except flotation of marginal root mats, BR18-0711-01C (1 ex., SEMC); Ji-Parana (27 km SW), Rio Urupa, rock pools along margins of river, -11.03618, -62.14465, 135 m, leg. Short, 10.vii.2018, rock pools along margins of river, BR18-0710-01A (1 ex., SEMC, DNA voucher SLE2332).

##### Differential diagnosis.

The dorsal coloration, shape of the elevation of the mesoventrite, area of pubescence on the metafemur and degree of impression of the ground punctation of *Notionotusgiraldoi* are very similar to *N.dilucidus*, *N.mexicanus*, *N.tricarinatus*. It can be distinguished only by its aedeagus, including the unique shape of the parameres with a depression of the inner margin, as well as by the abrupt narrowing in the midlength of the median lobe (Fig. 7D).

##### Description.

***Size and form***: Body length 1.7–1.9 mm. Body form elongate oval, convex in lateral view (Fig. 4I). ***Color and punctation***: Dorsally yellow, head yellow; pronotum paler than elytra, with two small black round spots along posterior margin (Fig. 4I). Ventrally brown; maxillary palps, mouthparts, antennae (antennal club slightly darker) and legs yellow. Clypeus and labrum with dense, fine, and weakly impressed ground punctation (punctures separated by 2 × their width); pronotum and elytra with ground punctation fine, weakly impressed and sparser than on head (punctures separated by 3 × their width). ***Head***: Clypeus and labrum shallowly emarginate anteromedially, lateral margins of the labrum bearing setae. ***Thorax***: Prosternum carinate medially, strongly raised, pointing anteriorly and acute. Elevation of mesoventrite with one transversal ridge, elevated medially, lateral sides concave; longitudinal ridge sharp, the point where the two ridges merged rounded and obtuse (e.g., Fig. 10A, B); elevation flat in lateral view; mesoventrite with triangular shape in ventral view. Metaventrite convex in the median region, pubescent with narrow glabrous patch on the medial and posterolateral area, medial region patch drop-shaped; anterior margin extending to mesoventrite elevation. Metafemora densely covered with hydrofuge pubescence on basal three-quarters. ***Abdomen***: Abdominal ventrites very densely pubescent. Aedeagus (Fig. 7D) with basal piece nearly the same length of a paramere. Base of the parameres slightly narrower than the base of the median lobe; outer margins sinuate, inner margins depressed in the midlength, depression extending to apex without reaching it; apex of parameres wide and blunt. Median lobe length almost equal to the parameres, wide at basal region, narrowing abruptly in the midlength, apical third slender, narrow, and rounded.

##### Etymology.

L. M. González-Rodríguez names this species in honor of Juan José Giraldo Gutiérrez in gratitude for the encouragement and support in her career.

##### Distribution.

Known from several localities in the Brazilian States of Rondonia and Mato Grosso do Sul (Fig. 14).

##### Life history.

This species was collected along the margins of two adjacent streams, one with a sandy bottom and one with a rocky bottom (Fig. 13A). Specimens were collected by agitating the sand and detritus along the stream margin as well as washing root mats in tubs of water.

#### ﻿ Notionotus liparus

Taxon classificationAnimaliaColeopteraHydrophilidae

Spangler, 1972

C6FB4C8F-F4BD-572D-8E23-E641A3FBE08A

Figs 2A–C , 7A , 11A, B , 14

Notionotus liparus Spangler, 1972: 144.

##### Type material examined.

***Holotype* (male)**: “VENEZUELA/ Bar., 24 Km. NW/ Barinitas/II-23-1969/P.&P. Spangler”, “Collected with/ 207 Oocyclus”, “HOLOTYPE/ Notionotus/liparus/P.J. Spangler” (USNM).

##### Additional material examined

**(139 exs.). Venezuela: Aragua State**: Henri Pittier National Park, Río Curucuruma, 10°21.070'N, 67°34.920'W, 11.i.2006, leg. A.E.Z. Short, waterfall/seep, AS-06-023 (75 exs., MIZA, SEMC, including DNA Voucher MSC1820); Henri Pittier National Park, Río Castaño Regresiva del Diablo, 10.35669°N, 67.60645°W, 6.i.2009, leg. A.E.Z. Short, log in stream, VZ09-0106-01A (5 exs., SEMC); same data except seeps/wet rock, VZ09-0106-01B (19 exs., SEMC); Henri Pittier National Park, Ranch Grande, 10.i.2006, leg. Short, stream and seep at Toma, AS-06-021, (8 exs., SEMC), same data except 2.vii.2020, VZ10-0702-01A (1 ex., SEMC, DNA voucher SLE2111). **Barinas State**: 13 km NW Barinitas, 8°48.424'N, 70°31.139'W, 992 m, 24.i.2012, leg. A. Short & Gustafson, seepage by road, VZ12-0124-02A (22 exs., SEMC, including DNA voucher SLE2123); same data except small stream pool, VZ12-0124-02B (1 ex., SEMC). **Mérida State**: ca. 2.5 km S. La Azulita, 8°44.335'N, 70°37.131'W, 842 m, 28.i.2012, leg. G.T. Gustafson, stream pools, VZ12-0128-02B (1 ex., SEMC); ca. 12 km, SE of Santo Domingo, 8°51.933'N, 70°37.131'W, 1682 m, 22.i.2012, leg. Short & Arias, wall seep 1, VZ12-0122-03A (7 exs., SEMC, including DNA voucher SLE2124). **Trujillo State**: ca. 3 km NE Laguna Agua Negro, 9°19.371'N, 70°9.303'W, 1770 m, 21.i.2009, leg. Short, García & Camacho, small mountain stream w/ detritus, VZ09-0121-03x (1 ex., SEMC).

##### Differential diagnosis.

*Notionotusliparus* can be recognized by the distinct black coloration among the other dark (reddish brown) species such as *N.brunbadius*, *N.parvus* and *N.retusus*, also for being the only dark species in the *liparus* group (Fig. 2A). It can also be differentiated by sharply marked punctation of the pronotum and elytra. Moreover, the outer margin of the parameres is sinuate and the apical third is slim and tapered. It is the only species in the *liparus* group with the apex of the median lobe acute.

##### Description.

***Size and form***: Body length 1.6–1.8 mm. Body form elongate oval, convex in lateral view (Fig. 2A). ***Color and punctation***: Dorsally black, with lateral margins of the pronotum and elytra reddish brown (Fig. 2A). Ventrally reddish brown, except for black abdominal ventrites; maxillary palps and antennae yellow (antennal club slightly darker) (Fig. 2B). Clypeus and labrum with dense, coarse, and moderately impressed ground punctation (punctures separated by 2 × their width); pronotum and elytra ground punctation dense, coarse, and moderately impressed and sparser than on head (punctures separated by 3 × their width). ***Head***: Clypeus and labrum shallowly emarginate anteromedially, lateral margins of the labrum bearing setae. ***Thorax***: Prosternum carinate medially, strongly raised, pointing anteriorly and acute. Elevation of mesoventrite with one transversal ridge, elevated medially, lateral sides concave; longitudinal ridge narrowed anteriorly and broadening posteriorly, the point where the two ridges merged blunt (e.g., Fig. 10A, B); elevation concave in lateral view; mesoventrite with triangular shape in ventral view. Metaventrite convex in the median region, pubescent with narrow glabrous patch on the medial and posterolateral area; anterior margin extending to mesoventrite elevation. Metafemora with dense hydrofuge pubescence along basal three-quarters of the anterior margin and along basal one-quarter of posterior margin, then apical half of the posterior margin with sparse setae (Fig. 2B). ***Abdomen***: Abdominal ventrites very densely pubescent. Aedeagus (Fig. 7A) with basal piece 1.3 × the length of a paramere. Base of the parameres slightly narrower than the base of the median lobe; outer margin sinuate, inner margin nearly straight, tapering along apical third, parameres thin and rounded at apex. Median lobe shorter than the parameres, broad at the base and gradually widening to the apex, with the apex acute.

##### Distribution.

This species is widespread in the Mérida Andes and Coastal Mountains of Venezuela (Fig. 14). Originally described from localities in the Venezuelan states of Barinas and Mérida (Spangler 1972, it was later recorded from the state of Aragua (García 2000). Here, we report additional localities in these three states as well as report it from the state of Trujillo for the first time.

##### Life history.

This species is found in rock seepage habitats and wet rocks adjacent to waterfalls (Fig. 11A, B). Occasionally it is found in the pools that form at the best of these habitats.

##### Remarks.

We sequenced four specimens from three Venezuelan states across the range of this species (Aragua, Barinas, and Mérida). The sequences are nearly identical (Fig. 1), supporting the concept of a widespread species in the Mérida Andes and Coastal mountains.

#### ﻿ Notionotus mexicanus

Taxon classificationAnimaliaColeopteraHydrophilidae

Perkins, 1979

ED05D427-A3D4-5C48-9515-53F49EDB5A8B

Figs 2D–F , 7B , 14

Notionotus mexicanus Perkins, 1979: 306.

##### Type material examined.

***Holotype* (male)**: “MEXICO, Oaxaca, 8 mi. E./Tapanatepec, tropical/stream with lg. boulders/3-VII-1974/ME&PD Perkins”, “Type No/76326/U S N M”, “HOLOTYPE/Notionotus/mexicanus/P.D.Perkins” (USNM).

##### Differential diagnosis.

*Notionotusmexicanus* is very similar morphologically to *N.tricarinatus* sharing characters such as body length, yellow dorsal coloration, pronotum and elytra with fine ground punctation, and elevation of the mesoventrite with a transversal and a longitudinal ridge. It can be distinguished by the shape of the aedeagus, specifically the shape of the parameres: inner margins straight and sinuate reaching the apex, parameres narrowing along apical third, and narrower than *N.tricarinatus*.

##### Description.

***Size and form***: Body length 1.8 mm. Body form elongate oval, convex in lateral view (Fig. 2D). ***Color and punctation***: Dorsally yellow, head mostly yellow, frons pale brown; pronotum with two small black round spots along posterior margin (Fig. 2D). Ventrally brown; maxillary palps, mouthparts, antennae, pro and meso legs yellow, meta legs pale brown (Fig. 2E). Clypeus and labrum with dense, fine, and weakly impressed ground punctation (punctures separated by 2 × their width); pronotum and elytra ground punctation fine, weakly impressed and sparser than on head (punctures separated by 3 × their width). ***Head***: Clypeus and labrum shallowly emarginate anteromedially, lateral margins of the labrum bearing setae. ***Thorax***: Prosternum carinate medially, strongly raised, pointing anteriorly and acute. Elevation of mesoventrite with one transversal ridge, elevated medially, lateral sides concave; longitudinal ridge sharp, the point where the two ridges merged rounded and obtuse (e.g., Fig. 10A, B); elevation concave in lateral view; mesoventrite with triangular shape in ventral view. Metaventrite convex in the median region, pubescent with narrow glabrous patch on the medial and posterolateral area; anterior margin extending to mesoventrite elevation. Metafemora densely covered with hydrofuge pubescence on basal three-quarters (Fig. 2E). ***Abdomen***: Abdominal ventrites very densely pubescent. Aedeagus (Fig. 7B) with basal piece 1.1 × the length of a paramere. Base of the parameres broader than the base of the median lobe; outer margin slightly convex along basal two-thirds, then slightly sinuate apically, inner margins nearly straight along basal two-thirds and then sinuate apically; apex of parameres rounded. Median lobe shorter than the parameres, approximately triangular, with acute apex.

##### Distribution.

Only known from the type locality in Mexico (Fig. 14).

##### Life history.

The type series was collected “from plant debris which had become trapped between stones in a rapid stream” (Perkins 1979).

#### ﻿ Notionotus tricarinatus

Taxon classificationAnimaliaColeopteraHydrophilidae

Perkins, 1979

756E9E93-6B7A-51C4-B06D-1606A8AC451A

**Figure 6.**
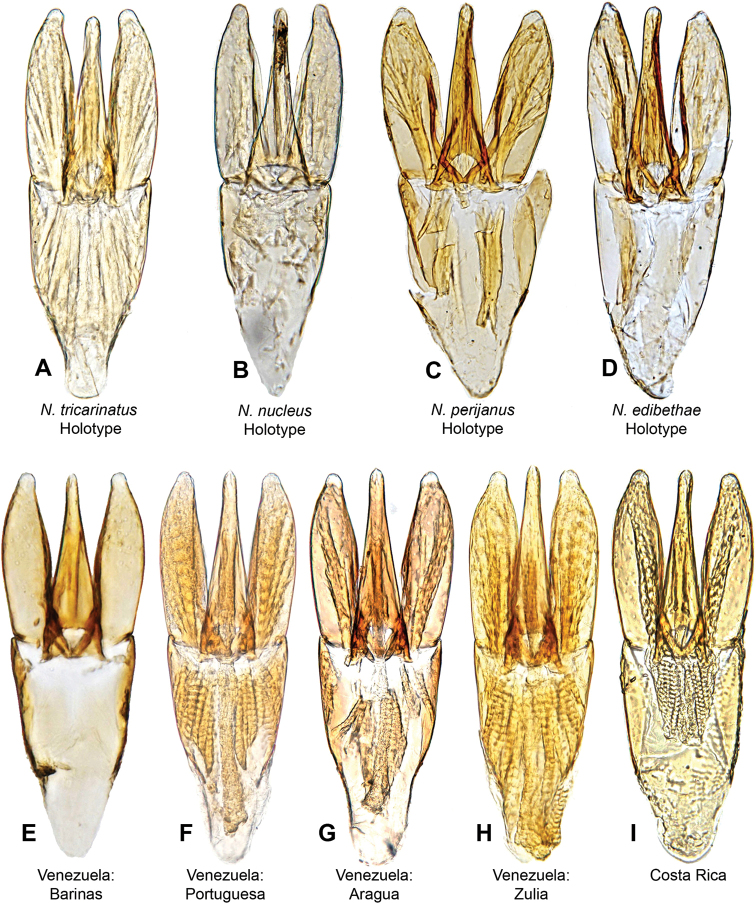
Aedeagi of *Notionotustricarinatus***A***N.tricarinatus* (holotype) **B***N.nucleus* (holotype) **C***N.perijanus* (holotype) **D***N.edibethae* (holotype) **E** specimen from Barinas State, Venezuela **F** specimen from Portuguesa State, Venezuela **G** specimen from Aragua State, Venezuela **H** specimen from Zulia State, Venezuela **I** specimen from Costa Rica.

Figs 3A–I , 6A–I , 11C, D , 14

Notionotus tricarinatus Perkins, 1979: 309.

##### Type material examined.

***Holotype* (male)**: “PANAMA, C.Z./Albrook Forest Site/ground, 22-III-/1968, R.S. Hutton”, Type No/76324/U S N M”, “HOLOTYPE/Notionotus/tricarinatus/P.D. Perkins” (USNM).

#### ﻿ Notionotus edibethae

Taxon classificationAnimaliaColeopteraHydrophilidae

García, 2000: 250. syn. nov.

5B031800-DB29-5D6A-98A1-1BDA8FD35CD2

##### Type material examined.

***Holotype* (male)**: “VENEZUELA. Trujillo/Mcpo. Rafael Rangel/La Guaira, Quebrada la/amarilla, 530 m./24-VIII-1997/Det. M. García, 199”, “Col:/M. García/J. Camacho/E. Gomez”, “Holotipo ♂/ Notionotus/edibethae/ Dcrip. M. García, 1997” (MALUZ). ***Paratypes* (2 exs.)**: “VENEZUELA: Trujillo/ Mcpo. Rafael Rangel/ La Gira, Quebrada la/ amarilla, 530 m./ 24-VIII-1997/ Det. M. García, 199”, “Col/ M. Garcia/ J. Camacho/”, “Paratipo ♀/ Notionotus/ edibethae/ Dcrip. M. García, 1997” (1 ex. SEMC); “VENEZUELA, Trujillo,/ Mcpo. Rafael Rangel, La/ Gira, Qda. La Amarilla,/ 520 m 20–22 / V / 1995/ Trampa Interceptación/”, “Colectores:/ J. Camacho/ M. García/”, “Paratipo ♂ / Notionotus/ edibethae/ Dcrip. M. García, 1997” (1 ex., SEMC). The labeled holotype is an undissected male with the aedeagus visible and still attached to the abdomen. We also examined a permanent genitalia slide that had been presumed to be from holotype and is labeled as this species.

#### ﻿ Notionotus nucleus

Taxon classificationAnimaliaColeopteraHydrophilidae

Perkins, 1979: 308. syn. nov.

E4789778-FFC7-521B-992A-1BBDB39C974A

##### Type material examined.

***Holotype* (male)**: “GUATE., Alta Verapaz/5 mi. W. La Tinta/small tropical brook/6-VI-1974, ME&PD Perkins”, “Type No/76325/U S N M”, “HOLOTYPE/Notionotus/nucleus/P.D. Perkins” (USNM).

#### ﻿ Notionotus perijanus

Taxon classificationAnimaliaColeopteraHydrophilidae

García, 2000: 252. syn. nov.

5F0EB05A-0AD2-5303-AF59-1B6F6E87EF8E

##### Type material examined.

***Holotype* (male)**: [only the permanent slide mount of the aedeagus was examined] (MALUZ). As this species was described only from one male and one female specimen, we presume this slide is of the holotype specimen (Fig. 6C).

##### Additional material examined

**(173 exs.). Costa Rica: Cartago Province**: Tapanti National Park, Building by Río Villegas, 29.v.2006, leg. A.E.Z. Short, HG-vapor light, AS-06-066 (5 exs., SEMC, INBio, including DNA voucher SLE2397). **Panama: Panama province**: Barrio Colorado, 9°11'N, 79°51'W, 40 m, 22–25-VI-2000, leg. S. Chatzimanolis, flight intercept trap, PAN1C00 022 (3 exs., SEMC); same data except PAN1C00 024 (5 exs., SEMC); same data except PAN1C00 025 (2 exs., SEMC); same data except PAN1C00 033 (1 ex., SEMC); same data except PAN1C00 0234 (1 ex., SEMC); same data except PAN1C00 014 (1 ex., SEMC); same data except 07-VI-1994, leg. D. Banks (2 exs., SEMC); same data except 08-VIII-1994, leg. D. Banks (1 ex., SEMC), same data except 08-VIII-1994, leg. D. Banks (1 ex., SEMC); same data except 04-VIII-1994, leg. D. Banks (1 ex., SEMC); same data except 01-VIII-1994, leg. D. Banks (1 ex., SEMC); Old plantation Rd. 6.9 km S Gamboa, 09°05'N, 79°40'W, 80 m, 04–07-VI-1995, leg. J, Ashe & R. Brooks, #137 flight intercept trap (1 ex., SEMC), same data except 07–22-VI-1995, #266 flight intercept trap (1 ex., SEMC); Colón, Parque Nacional Soberanía, Pipeline Rd km 6.1, 09°07'N, 79°45'W, 40 m, 07–21-VI-1995, leg. J, Ashe & R. Brooks, #265 flight intercept trap (2 exs., SEMC); Colón, Escobal & Piña Rds, 14 km N jct. 02–11-VI-1996, leg. J, Ashe & R. Brooks flight intercept trap PAN1AB96 181B (1 ex., SEMC). **Venezuela: Aragua State**: Henri Pittier Natural Park Río Cumboto, 10.39376°N, 67.79597°W, 130 m, 4.i.2009, leg. Short, García & Miller, river side pools, VZ09-0104-02B (18 exs., SEMC, including DNA voucher SLE2381); Río La Trilla 10.37319°N, 67.74250°W, 295 m, leg. Short, García & Miller, pools, VZ09-0104-01A (1 ex., SEMC). **Barinas State**: Río Santa Barbara, E. Santa Barbara. 7°50.028N, 71°11.188W, 177 m, 26.i.2012, leg. Short, Arias, Gustafson, sandy sidepool in floodplain, VZ12-0126-01B, (1 ex., SEMC). **Portuguesa State**: Trib. of Río Guanare, S. Biscucuy, 9°14.457'N, 69°55.994'W, 370 m, 19.i.2009, leg. Short, García & Miller, gravel stream, VZ09-0119-03X (15 exs., SEMC, including DNA vouchers SLE2391 and SLE2392). **Zulia State**: Perijá Natural Park Tukuko: Río Manantial, 9°50.490'N, 72°49.310'W, 270 m, 29.i.2009, leg. Short, García & Camacho, gravel margin, VZ09-0129-01A (91 exs., MIZA, SEMC, including DNA vouchers SLE1112 and SLE2371); same data except 29.i.2009, leg. Short, García & Miller, detrital pool, VZ09-0129-01B (1 ex., SEMC); same data except 22.ix.2007, leg. A.E.Z. Short, rock pools/margin, AS-07-020b (8 exs., SEMC); Toromo, 10°03.058'N, 72°49.974'W, 435 m, 31.xii.2005, leg. A.E.Z. Short, small stream and seep, AS-06-001 (6 exs., SEMC); same data except 28.i.2009, detrital pool, VZ09-0128-01A (3 exs., SEMC).

##### Differential diagnosis.

See differential diagnosis for *Notionotusmexicanus*.

##### Description.

***Size and form***: Body length 1.6–1.9 mm. Body form elongate oval, strongly convex in lateral view (Fig. 3A). ***Color and punctation***: Dorsally yellow, head mostly pale brown or yellow, frons brown or dark brown; pronotum paler than elytra, with two small black round spots along posterior margin (Fig. 3A, D, G). Ventrally brown; maxillary palps, mouthparts, antennae yellow (antennal club slightly darker), legs pale brown (Fig. 3B, E, H). Clypeus and labrum with dense, fine, and weakly ground punctation (punctures separated by 2 × their width); pronotum and elytra ground punctation fine, weakly impressed and sparser than on head (punctures separated by 3 × their width). ***Head***: Clypeus and labrum shallowly emarginate anteromedially, lateral margins of the labrum bearing setae. ***Thorax***: Prosternum carinate medially, strongly raised, pointing anteriorly and acute. Elevation of mesoventrite with one transversal ridge, elevated medially, lateral sides concave; longitudinal ridge sharp and broadening posteriorly almost to the end, the point where the two ridges merged rounded and obtuse (e.g., Fig. 10A, B); elevation concave in lateral view; mesoventrite with triangular shape in ventral view. Metaventrite convex in the median region, pubescent with narrow glabrous patch on the medial and posterolateral area; anterior margin extending to mesoventrite elevation. Metafemora densely covered with hydrofuge pubescence on basal three-quarters (Fig. 3B, E, H). ***Abdomen***: Abdominal ventrites very densely pubescent. Aedeagus (Fig. 6A–I) basal piece 1.2 × the length of a paramere. Parameres broad, base wider than the base of the median lobe, outer and inner margins convex, pinched at the apex, broad and rounded apex. Length of the median lobe can vary (median lobe as long as the parameres (Fig. 6F, I), slightly shorter (Fig. 6A, B) or longer than the parameres (Fig. 6H), median lobe with triangular shape, wide at base and gradually tapering to apical third, with rounded apex.

##### Distribution.

Known from Guatemala, Costa Rica, Panama, and Venezuela (Fig. 14).

##### Life history.

This species is found along the margins and in leaf packs of streams in the mountains and foothills of the Northern Andes and Central America. It prefers gravelly or rocky streams, especially in the foothills where it may sometimes be abundant (Fig. 11C, D).

##### Remarks.

We examined more than 155 specimens from a dozen localities of this species from Guatemala to several chains of the Venezuelan Andes. Although there are subtle variations in the apex of the aedeagal parameres, this variation is relatively small and not correlated to geography, other morphological characters, or molecular data. These subtle variations in paramere shape likely explain why this species has been described four times, once each from Guatemala and Panama (Perkins 1979) and twice from Venezuela (García 2000). Three of these four species were described from single collecting events. However, with significantly more material available to us for this study from a range of additional localities, it is apparent these differences in paramere shape are more easily considered as intraspecific variation in a widespread, common species than as indicative of species boundaries. This hypothesis is also supported by available DNA evidence: we sequenced specimens from Costa Rica, the Serranía de Perijá, the Mérida Andes, as well as the Coastal mountains of Venezuela. All specimens form a clade (Fig. 1) with a maximum pairwise divergence in COI of 3.9% (although we only had COI data from specimens from several Venezuelan localities). However, 28S sequence data from specimens from Venezuela and Costa Rica are identical, further supporting the concept of a single, widespread species. In addition, specimens throughout its range were found in very similar habitats: leaf packs or detrital margins of streams with a gravel or rocky substrate

As both *N.nucleus* and *N.tricarinatus* were proposed in the same work (Perkins 1979), we use our authority as first revisors (ICZN article 24.2.2) to give precedence to *N.tricarinatus* as the valid name for this species.

#### ﻿ Notionotus vatius sp. nov.

Taxon classificationAnimaliaColeopteraHydrophilidae

BCB825EF-8025-5E7E-BCD5-C4220D99CE9A

http://zoobank.org/CD665789-B94F-4C19-AAE1-83B7ACCE9413

**Figure 7.**
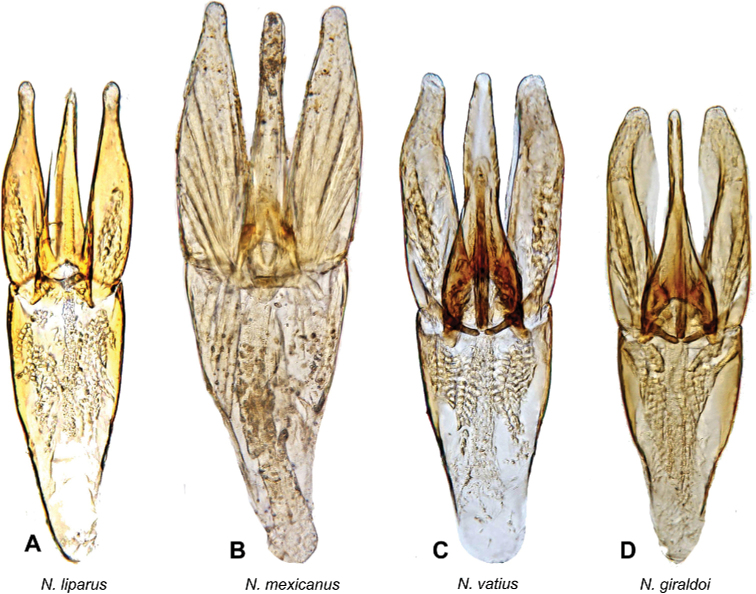
Aedeagi of *Notionotusliparus* species group **A***N.liparus* (non-type specimen) **B***N.mexicanus* (holotype) **C***N.vatius* (holotype) **D***N.giraldoi* (holotype).

Figs 4H , 7C , 13B , 14

##### Type material.

***Holotype* (male)**: “BRAZIL: Mato Grosso do Sul/ -20.72281°, -55.69127°; 225 m/Aquidauana (c. 27 km S) on/MS-174; leg. Hamada & team;/27.vi.2018; seepage & debris nr./stream margin; BR18-0627-01E” (INPA). ***Paratypes* (38 exs.): Brazil: Mato Grosso do Sul State**: Same data as holotype (19 exs., INPA, SEMC, including DNA Voucher 2324); Aquidauana on plateau (ca. 15 km E), -20.4509, -55.6218, 380 m, 22.vi.2018, leg. Hamada & team, detritus and washing roots at margin on rock, BR18-0622-03D (6 exs., SEMC, including DNA voucher SLE2327); Corumbá (ca. 27 km SE) by mountains, -19.28382, -57.57506, 146 m, 24.vi.2018, leg. Hamada & team, stream margin and leaf packs, BR18-0624-01A (1 ex., SEMC); Rio Bento Gomes (Pantanal), Campo Allegre I, 15°45'S, 56°33'W, 1993–1994, leg. E. Stuhr, spring-fed brook, (10 exs., NMW, SEMC). **Bahia**: Morro do Chapéu, Cachoeira Domingos Lopes, -11.55965, -40.90635, 675 m, 24.ii.2018, leg. Benetti & team, blackwater river and waterfall, BR18-0224-02A (1 ex., SEMC, DNA Voucher SLE2104); Livramento de Nossa Senhora, NE on BR-148, -13.6212, -41.81908, 536 m, 27.ii.2018, leg. Benetti & team, stream margins, BR18-0227-01A (1 ex., SEMC, DNA Voucher SLE2385).

##### Differential diagnosis.

Among the species of *liparus* group, *Notionotusvatius* can be recognized by the brown dorsal coloration, and quite unique color pattern of the head frons and medial region of the clypeus dark brown, lateral side of the clypeus pale brown. In addition, the shape of aedeagus, especially the apex of the parameres is broad, blunt, and pointing slightly outwards, and the gonopore has rounded shape and it is situated at midlength of median lobe.

##### Description.

***Size and form***: Body length 1.7–2.3 mm. Body form elongate oval, strongly convex in lateral view (Fig. 4H). ***Color and punctation***: Dorsally brown, head mostly brown, frons and medial region of the clypeus dark brown, lateral side of the clypeus pale brown; pronotum pale brown with two small black round spots along posterior margin, elytra dark brown (Fig. 4H). Ventrally dark brown; maxilla, maxillary palps, antennae (antennal club slightly darker) yellow, legs pale brown. Clypeus and labrum with dense, fine, and weakly impressed ground punctation (punctures separated by 2 × their width); pronotum and elytra ground punctation fine, weakly impressed and sparser than on head (punctures separated by 3 × their width). ***Head***: Clypeus and labrum shallowly emarginate anteromedially, lateral margins of the labrum bearing setae. ***Thorax***: Prosternum carinate medially, strongly raised, pointing anteriorly and acute. Elevation of mesoventrite with one transversal ridge, elevated medially, lateral sides concave; longitudinal ridge sharp, the point where the two ridges merged rounded and obtuse (e.g., Fig. 10B); elevation flat in lateral view; mesoventrite with triangular shape in ventral view. Metaventrite convex in the median region, pubescent with narrow glabrous patch on the medial and posterolateral area, medial region patch drop-shaped; anterior margin extending to mesoventrite elevation. Metafemora densely covered with hydrofuge pubescence on basal three-quarters. ***Abdomen***: Abdominal ventrites very densely pubescent. Aedeagus (Fig. 7C) with basal piece nearly the same length as a paramere. Base of the parameres slightly narrower than the base of the median lobe; outer and inner margins sinuate; apex of parameres wide and blunt, pointing outwards. Median lobe of the same length as the parameres, wide at basal region, narrowing in the midlength, then widening along at apical third, apex rounded; gonopore with rounded shape and situated at midlength of median lobe.

##### Etymology.

The name is derived from the Latin word *vatius* meaning bent outwards, after the form of the parameres slightly pointing outwards of the aedeagus.

##### Distribution.

This species is known from several localities in Bahia and Mato Grosso do Sul States in Brazil (Fig. 14).

##### Life history.

This species was collected on seepages and along the margins of rocky streams (Fig. 13B).

##### Remarks.

Although the known localities of this species are widely dispersed in Brazil, they are from similar habitats at similar elevations on the Brazilian Shield. Moreover, the specimens from Bahia and Mato Grosso do Sul states are less than 3% divergent in uncorrected pairwise distances in COI.

###### *Notionotuslohezi* species group

**Diagnosis.** The *lohezi* species group can be distinguishable by the presence of three ridges in the elevation of the mesoventrite, two transverse ridge and one longitudinal (Fig. 10C, D); the basal piece is shorter than the parameres, the length of the median lobe is shorter than the parameres.

#### ﻿ Notionotus bicolor sp. nov.

Taxon classificationAnimaliaColeopteraHydrophilidae

8F682BCC-D00D-5DED-9259-04BB1E03B143

http://zoobank.org/7954CB9D-66E9-492F-865E-90BA4991ADF3

**Figure 8.**
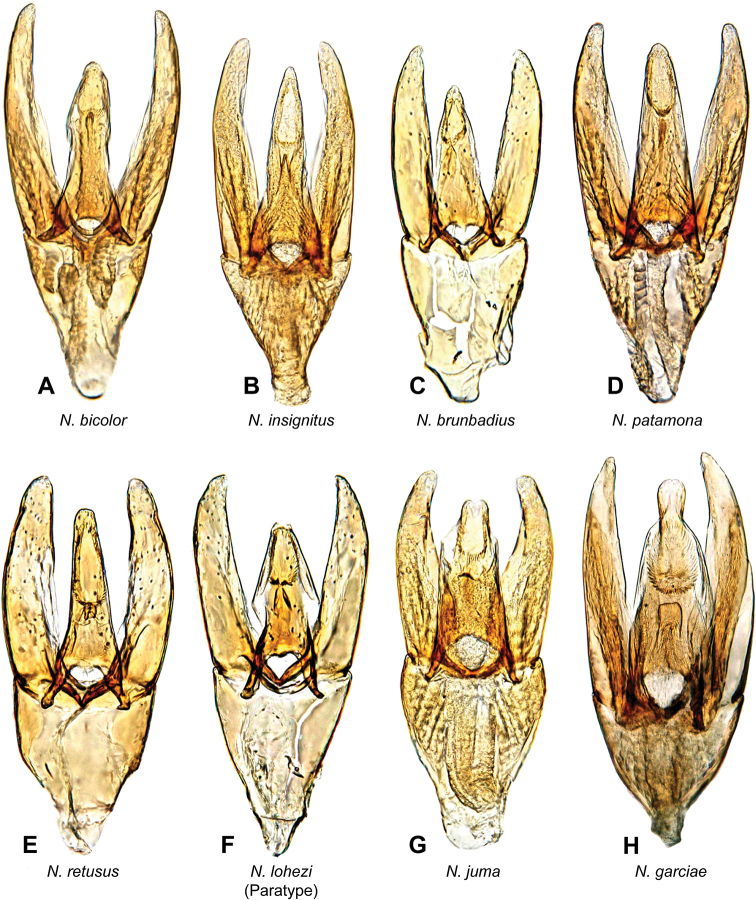
Aedeagi of *Notionotuslohezi* species group **A***N.bicolor* (holotype) **B***N.insignitus* (holotype) **C***N.brunbadius* (holotype) **D***N.patamona* (holotype) **E***N.retusus* (holotype) **F***N.lohezi* (paratype) **G***N.juma* (holotype) **H***N.garciae* (holotype).

Figs 4A , 8A , 10D , 12D , 15

##### Type material examined.

***Holotype* (male)**: “SURINAME: Sipaliwini District/ 4.42313°N, 57.19198°W, 104 m/ Kabalebo Nature Resort/ Moi Moi Creek; 10–14.iii.2019/ leg Short & Baca small seeps/ SR19-0310-01F” (NZCS). ***Paratypes* (88 exs.): Suriname: Sipaliwini District**: Same data as holotype (8 exs., SEMC); Kabalebo Nature Resort: Charlie Falls, 4.38302°N, 57.21161°W, 174 m, 11.iii.2019, leg. Short, Baca and class, rocks pools in creekbed, SR19-0311-01A (55 exs., NZCS, SEMC, including DNA vouchers SLE1810 and SLE2120); same data except leg. Short, Seepage, SR19-0311-01B (14 exs., SEMC); same data except leg. Baca, side pools in gravel in creekbed, SR19-0311-01E (2 exs., SEMC); Kabalebo Nature Resort Moi Moi creek, 4.42313°N, 57.19198°W, 104 m, 10–14.iii.2019 leg. Short, Baca and class, margin of seepage, SR19-0310-01E (2 exs., SEMC); same data except leg. Baca, margin of stream pool with root mats, SR19-0310-01L (1 ex., SEMC); same data except leg. Short and class, small trickle on rocks w/detritus, SR19-0310-01M (4 exs., SEMC); CSNR: Tafelberg Summit, near Caiman Creek Camp, 3°53.942'N, 56°.10.849'W, 733 m, 19.viii.2013, leg. Short and Bloom, margins and leaf packs in Caiman Creek, SR13-0819-05D (2 exs., SEMC).

##### Differential diagnosis.

*Notionotusbicolor* shares the bicolorous dorsal coloration that can also be observed in *N.garciae*, *N.patamona*, and *N.lohezi*. It can be recognized by the shape of the aedeagus, the median lobe is much shorter than in *N.insignitus* (Fig. 8B) and *N.patamona* (Fig. 8D), and slightly longer than in *N.lohezi* (Fig. 8F). The margins of the median lobe are sinuate with a widening in the middle length, this differs in the other species where the margins are slightly straight. Moreover, the apex is slightly narrow and rounded, this differs in *N.insignitus* with acute apex, *N.patamona* with rounded and wide apex and *N.lohezi* with blunt apex.

##### Description.

***Size and form***: Body length 1.7–2 mm. Body form elongate oval, moderately convex in lateral view (Fig. 4A). ***Color and punctation***: Dorsally bicolor, head brown, frons dark brown; pronotum yellow with two small black round spots along posterior margin; elytra dark brown, elytra margins paler (Fig. 4A). Ventrally brown; maxillary palps, mouthparts, and antennae yellow (antennal club slightly darker), pro legs yellow, meso and meta legs pale brown. Clypeus and labrum with dense, fine, and weakly impressed ground punctation (punctures separated by 2 × their width); pronotum and elytra ground punctation fine, weakly impressed and sparser than on head (punctures separated by 3–4 × their width). ***Head***: Clypeus and labrum shallowly emarginate anteromedially, lateral margins of the labrum bearing setae. ***Thorax***: Prosternum carinate medially, strongly raised, acute and pointing anteriorly. Elevation of mesoventrite with two transversal ridges, elevated medially, lateral sides concave; longitudinal ridge broad anteriorly and sharp posteriorly, the point where the three ridges merged wide and blunt (Fig. 10D); elevation flat in ventral view; mesoventrite with triangular shape in ventral view. Metaventrite convex in the median region, pubescent with narrow glabrous patch on the medial and posterolateral area; anterior margin extending to mesoventrite elevation. Metafemora with dense hydrofuge pubescence along basal three-quarters of the anterior margin and sparse pubescence along the entire basal posterior margin. ***Abdomen***: Abdominal ventrites very densely pubescent. Aedeagus (Fig. 8A) with basal piece 0.7 × the length of a paramere. Base of the parameres slightly narrower than the base of the median lobe; outer margin convex, inner margins along basal two-thirds slightly convex, apical third straight; apex of parameres slightly acute, pointing inwards. Median lobe shorter than the parameres, wide at basal region, narrowing in the midlength, then sinuate, apex slightly acute; gonopore situated at the apex of median lobe.

##### Etymology.

The name derived from the Latin words *bi* meaning two and *color* meaning hue, referring to the dorsal coloration of the species. This species has yellow coloration in the pronotum and black in the elytra.

##### Distribution.

Known from two localities in central Suriname: Kabalebo and the summit of Tafelberg Tepui (Fig. 15).

##### Life history.

At Kabalebo, this species was collected in several streams, including both along the margin, rock pools with detritus in the creek bed, and in seepage habitats (Fig. 12D). At Tafelberg, the species was collected along a rocky creek margin with detritus.

#### ﻿ Notionotus bifidus sp. nov.

Taxon classificationAnimaliaColeopteraHydrophilidae

0D0CE7E5-2C3C-530E-95A1-195899958AFD

http://zoobank.org/AEAADA10-0A40-4AB9-B48E-FC27A126792B

**Figure 9.**
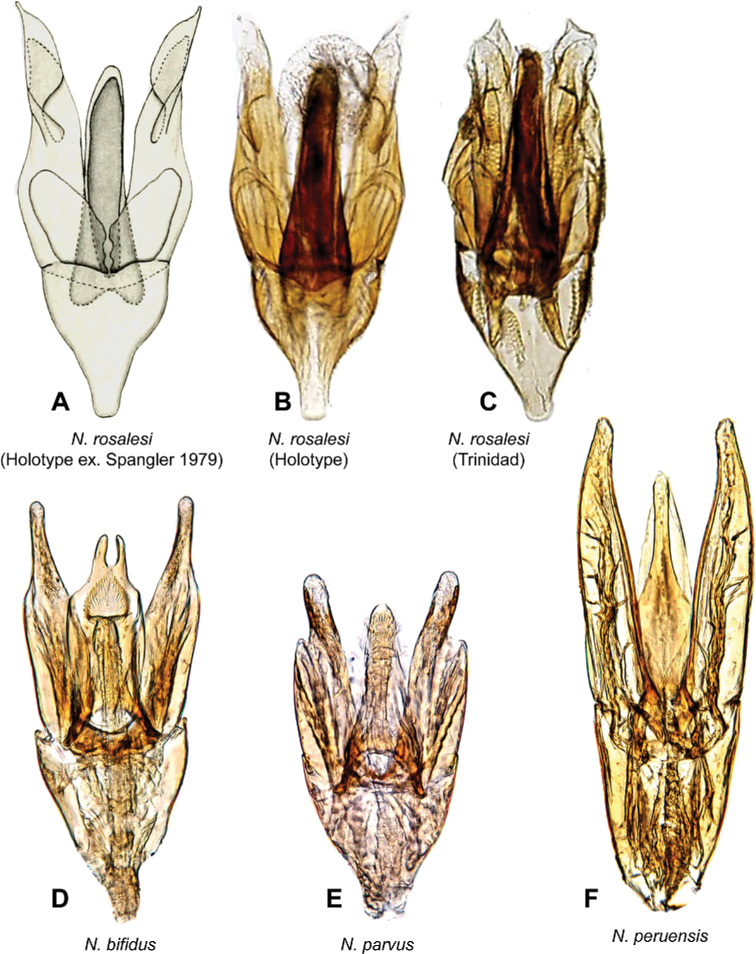
Aedeagi of *Notionotus* spp. **A–C***N.rosalesi***A** drawing from Spangler (1972)**B** holotype **C** specimen from Trinidad **D***N.bifidus***E***N.parvus***F***N.peruensis*.

Figs 4J , 9D , 12A , 14

##### Type material.

***Holotype* (male)**: “VENEZUELA: Amazonas State/ 5°23.207'N, 67°36.922'W, 125 m/ Tobogan de la Selva; 8.viii.2008/leg. A. Short, M. García, L. Joly/ AS-08-080b; old “tobogancito”/on seepage area w/detritus” (MIZA). ***Paratypes* (54 exs.): Venezuela: Amazonas State**: Same data as holotype (18 exs., SEMC, including DNA voucher SLE1113); same data except 14.i.2009, leg. A. Short, clumps of wet leaves on rock, VZ09-0114-01D (15 exs., SEMC, including DNA voucher SLE2369); same date except 14.i.2009, leg. K. Miller, detrital rock pools, VZ09-0114-01E (1 ex., SEMC); same data except 14.i.2009, leg. Short & Miller partly shaded wet rock w/algae, VZ09-0114-01G (20 exs., MIZA, SEMC).

##### Differential diagnosis.

*Notionotusbifidus* can be separated from all other species of *lohezi* group by being the only species in the group that present uniformly dorsal yellow coloration (Fig. 4J), the rectangular shape of the median lobe and its bifurcation at the apex, and the abrupt tapering of the parameres along apical third (Fig. 9D).

##### Description.

**Figure 10.**
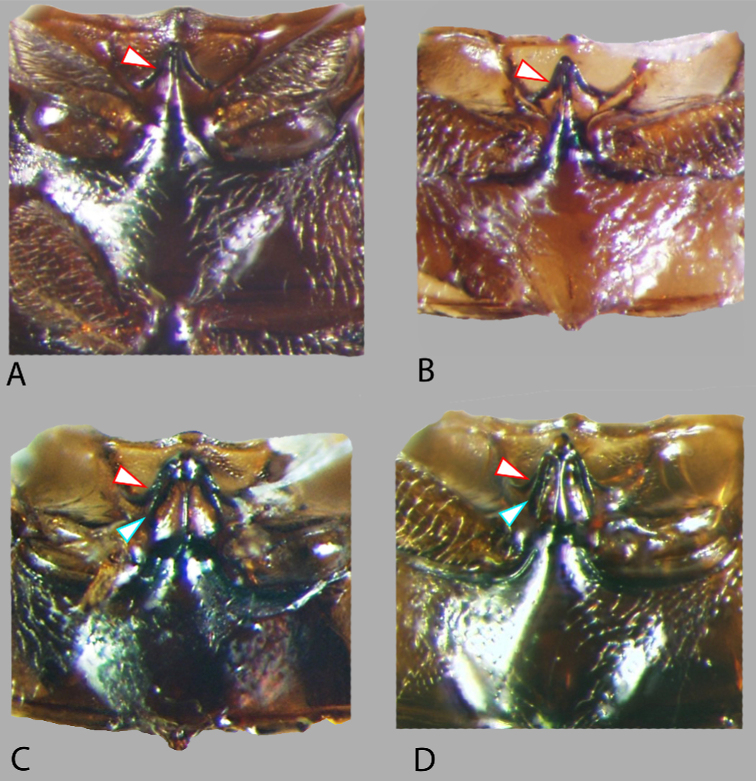
Mesoventral process of *Notionotus* spp. **A***N.dilucidus***B***N.tricarinatus***C***N.insignitus***D***N.bicolor*. Red marks pointing to transverse ridge in **A–D** blue marks pointing to second transverse ridge in **C, D**.

***Size and form***: Body length 1.5–1.7 mm. Body form elongate oval, moderately convex in lateral view (Fig. 4J). ***Color and punctation***: Dorsally yellow, head mostly dark brown, frons and medial region of the clypeus dark brown, lateral sides of clypeus yellow; pronotum yellow with two small dark brown round spots along posterior margin; elytra yellow (Fig. 4J). Ventrally dark brown; maxillary palps, mouthparts, and antennae yellow. Pro legs yellow, meso and meta legs pale brown. Clypeus and labrum with dense, fine, and weakly impressed ground punctation (punctures separated by 2 × their width); pronotum and elytra ground punctation fine, weakly impressed and sparser than on head (punctures separated by 3 × their width). ***Head***: Clypeus and labrum shallowly emarginate anteromedially, lateral margins of the labrum bearing setae. ***Thorax***: Prosternum carinate medially, strongly raised, acute and pointing anteriorly. Elevation of mesoventrite with two transversal ridges, elevated medially, lateral sides concave; longitudinal ridge broad anteriorly and sharp posteriorly, the point where the three ridges merged wide and blunt (e.g., Fig. 10C, D); elevation flat in ventral view; mesoventrite with triangular shape in ventral view. Metaventrite convex in the median region, pubescent with broad glabrous patch on the medial and posterolateral area; anterior margin extending to mesoventrite elevation. Metafemora with dense hydrofuge pubescence along basal three-quarters of the anterior margin and along basal one-quarter of posterior margin, then apical half of posterior margin with sparse setae. ***Abdomen***: Abdominal ventrites very densely pubescent. Aedeagus (Fig. 9D) with basal piece 0.6 × the length of a paramere. Base of the parameres narrower than the base of the median lobe; outer margins straight along basal two-thirds, then narrowing abruptly along apical third, inner margins convex along basal two-thirds and then apically slightly concave; apex of parameres rounded. Median lobe shorter than the parameres, approximately rectangular, narrow along apical fifth, apex bifurcated; gonopore drop-shaped and situated at apical fourth of median lobe.

##### Etymology.

The specific name comes from the Latin word *bifidus* meaning split into two parts, after the form of the median lobe “bifurcated apically” of the aedeagus.

##### Distribution.

Known only from the type locality in Venezuela (Fig. 14).

##### Life history.

This species was collected in seepage habitats that were covered with algae and detritus (Fig. 12A).

#### ﻿ Notionotus brunbadius sp. nov.

Taxon classificationAnimaliaColeopteraHydrophilidae

23339019-5334-5BC5-80D9-908E62640698

http://zoobank.org/23615726-EDE5-40AD-B408-8ED24AC30EDC

Figs 4C , 8C , 12C , 15

##### Type material.

***Holotyp*e (male)**: “BRAZIL: Amazonas, Manaus/-2.93079, -59.97514, 75 m/ Ducke Reserve/ leg. Short & team; stream margin/ & assoc. backwater swampy area/ 9–10.vi.2018; BR18-0609-03A”, “DNA VOUCHER/ Extraction #/ SLE-1553” (INPA). ***Paratypes* (4 exs.): Brazil: Amazonas State**: Same data as holotype (2 exs., SEMC); same data except Igarape Barro Branco, muddy pools in swampy area adjacent to stream, BR18-0609-02B (1 ex., SEMC, DNA voucher SLE2102); same data except by unnamed stream, water in palm fronds, BR18-0609-03B (1 ex., SEMC).

##### Differential diagnosis.

This species has a particular coloration pattern, dark reddish brown, which makes it easily distinguishable among the other species of the *lohezi* group. The shape of the parameres is slightly similar to *N.lohezi* (Fig. 8F) but the apex is less acute and bend much less inward. It can be separated by the triangular shape of the median lobe and acute apex (Fig. 8C).

##### Description.

***Size and form***: Body length 1.7–1.9 mm. Body form elongate oval, moderately convex in lateral view (Fig. 4C). ***Color and punctation***: Dorsally dark reddish brown, lateral margins of clypeus, pronotum and elytra yellow brown (Fig. 4C). Ventrally reddish brown, except for black abdominal ventrites; maxillary palps, mouth parts and antennae yellow (antennal club slightly darker). Clypeus and labrum with dense, fine, and weakly impressed ground punctation (punctures separated by 2 × their width); pronotum and elytra ground punctation fine, weakly impressed, and sparser than on head (punctures separated by 3–4 × their width). ***Head***: Clypeus and labrum shallowly emarginate anteromedially, lateral margins of the labrum bearing setae. ***Thorax***: Prosternum carinate medially, strongly raised, acute and pointing anteriorly. Elevation of mesoventrite with two transversal ridges, elevated medially, lateral sides concave; longitudinal ridge broad anteriorly and sharp posteriorly, the point where the three ridges merged wide and blunt (e.g., Fig. 10C, D); elevation flat in ventral view; mesoventrite with triangular shape in ventral view. Metaventrite convex in the median region, pubescent with narrow glabrous patch on the medial and posterolateral area; anterior margin extending to mesoventrite elevation. Metafemora with dense hydrofuge pubescence along three-quarters of the anterior basal margin and sparse pubescence along the posterior basal margin. ***Abdomen***: Abdominal ventrites very densely pubescent. Aedeagus (Fig. 8C) with basal piece 0.7 × the length of a paramere. Base of the parameres narrower than the base of the median lobe; outer margins slightly convex, inner margins straight; apex of parameres acute and pointing outwards. Median lobe shorter than the parameres, approximately triangular, wide at the basal region, then slightly narrowing to the apex, apex acute, gonopore oval in shape and situated at the apex of the median lobe.

##### Etymology.

The name is a combination of two Latin words *brun* meaning dark and *badius* meaning reddish brown, highlighting the distinguishable dark reddish brown color of this species.

##### Distribution.

Known only from the type locality near Manaus, Brazil (Fig. 15).

##### Life history.

The only known specimens were collected along the margin of a stream (Fig. 12C).

#### ﻿ Notionotus garciae sp. nov.

Taxon classificationAnimaliaColeopteraHydrophilidae

0F117129-1291-5924-B9FD-43329AF6AD68

http://zoobank.org/10EFC8E2-7C02-4F44-994B-54B3ABB388C8

Figs 4G , 8H , 13D , 15

##### Type material.

***Holotype* (male)**: “BRAZIL: Amazonas: Manaus/ -2.93079, -59.97514, 75 m/ Ducke Reserve, stream nr./ transect trail; leg. Short & team/ stream margins & leaf packs/ 9.vi.2018; BR18-0609-01A” (INPA). ***Paratypes* (8 exs.): Brazil: Amazonas State**: Same data as holotype (3 exs., SEMC); same data except 9–10.vi.2018, stream margin and associate backwater swampy area, BR18-0609-03A (1 ex., SEMC); same data except Igarape Barro Branco, 6.vi.2018, shallow pools, BR18-0606-02D (1 ex., SEMC); same data except Igarape Barro Branco, margin of stream, 9.vi.2018, BR18-0609-02A (2 exs., SEMC); Presidente Figueiredo (ca. 57 km E) on AM-240, -1.98826, -59.51618, 80 m, airport stream, 19.vi.2018, leg. Short, margin of creek, (1 ex., SEMC, DNA voucher SLE1900).

**Figure 11.**
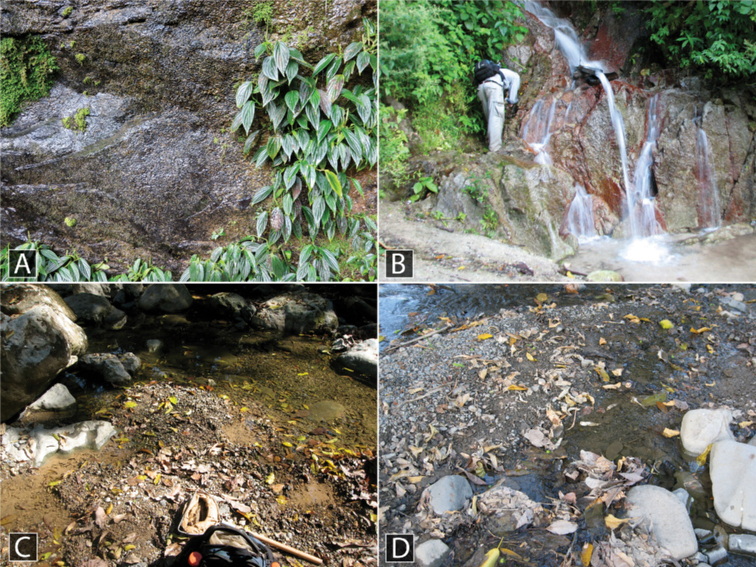
Habitat of *Notionotus* spp. in the Andean region of Venezuela **A** Venezuela, Barinas State, seepage habitat of *N.liparus* (collecting event VZ12-0124-02A) **B** Venezuela: Aragua, Henri Pittier National Park, seepage habitat of *N.liparus*, (collecting event AS-06-023) **C** Venezuela: Zulia, Rio Tukuko, habitat of *N.tricarinatus* (collecting event VZ09-0129-01A) **D** Venezuela, Portuguesa, tributary of the Rio Guanare, habitat of *N.tricarinatus* (collecting event VZ09-0119-03X).

##### Differential diagnosis.

*Notionotusgarciae* is very similar to *N.juma* in the bicolor dorsal coloration, the shape of the elevation of the mesoventrite and the pubescent area of the metafemora. It can be distinguished by the punctation of the pronotum, the elytra shallowly marked (in *N.juma* is moderately impressed, Fig. 8G), and the distinctive rounded and broad apex of the median lobe (Fig. 8H).

##### Description.

***Size and form***: Body length 1.7–1.8 mm. Body form elongate oval, convex in lateral view (Fig. 4G). ***Color and punctation***: Dorsally bicolor, head mostly brown, frons dark brown, posteromedial margin of the clypeus pale brown and lateral sides and anterior margin yellow; pronotum yellow with two small black round spots along posterior margin; elytra brown, lateral and posterior side of the elytra yellow (Fig. 4G). Ventrally dark brown; maxillary palps, mouthparts, and antennae yellow. Pro and meso legs yellow, meta legs pale brown. Clypeus and labrum with dense, fine, and weakly impressed ground punctation (punctures separated by 2 × their width); pronotum and elytra ground punctation weakly impressed, fine, and sparser than on head (punctures separated by 3 × their width). ***Head***: Clypeus and labrum shallowly emarginate anteromedially, lateral margins of the labrum bearing setae. ***Thorax***: Prosternum carinate medially, strongly raised, acute and pointing anteriorly. Elevation of mesoventrite with two transversal ridges, elevated medially, lateral sides concave; longitudinal ridge broad anteriorly and sharp posteriorly, the point where the three ridges merged wide and blunt (e.g., Fig. 10C, D); elevation flat in ventral view; mesoventrite with triangular shape in ventral view. Metaventrite convex in the median region, pubescent with narrow glabrous patch on the medial and posterolateral area; anterior margin extending to mesoventrite elevation. Metafemora with dense hydrofuge pubescence along basal three-quarters of the anterior margin and sparse pubescence along the apical posterior margin. ***Abdomen***: Abdominal ventrites very densely pubescent. Aedeagus (Fig. 8H) with basal piece 0.4 × the length of a paramere. Base of the parameres narrower than the base of the median lobe; outer margins nearly convex along basal two-thirds, then curved inwards along apex, inner margins nearly straight; apex of parameres acute. Median lobe shorter than the parameres, wide at basal region, narrowing in the midlength and then slightly broadening to apex, apex rounded and wide; gonopore ovate in shape and situated at apical fourth of median lobe.

##### Etymology.

This species is named after Andrea Lorena García Hernández curator at the Colección de Insectos de la Universidad del Quindío (CIUQ) in recognition to her passion and contribution of the knowledge of the insects, specially hydrophilids in Colombia.

##### Distribution.

This species is known from several collecting events at the Ducke Reserve near Manaus, Brazil (Fig. 15).

##### Life history.

This species was collected along the margins of small streams in dense forest (Fig. 13D).

#### ﻿ Notionotus insignitus sp. nov.

Taxon classificationAnimaliaColeopteraHydrophilidae

FB77835A-978C-5989-8931-5BBB32322C1F

http://zoobank.org/9B5D9358-AC4A-49D1-B9F6-44490E2FAA7C

**Figure 12.**
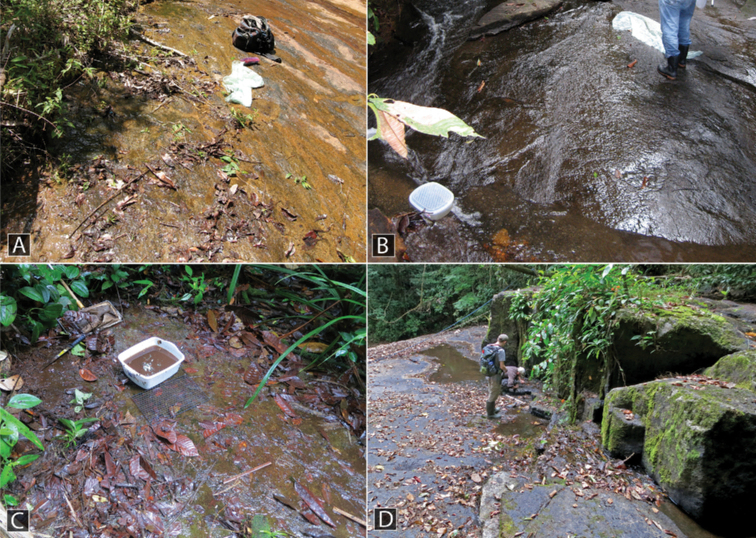
Habitat of *Notionotus* spp. **A** Venezuela: Amazonas State, type locality and habitat of *N.bifidus* (collecting event AS-08-080b) **B** Venezuela: Bolívar State: Type locality and habitat of *N.insignitus* (collecting event AS-08-058) **C** Guyana: Region 9: type locality and habitat of *N.patamona* (collecting event GY13-0318-01C) **D** Suriname: Kabalebo, habitat and type locality for *N.bicolor* (collecting event SR19-0310-01F).

Figs 4D , 8B , 10C , 12B , 15

##### Type material.

***Holotype* (male)**: “VENEZUELA: Bolívar State/ 6°2'10.5"N, 61°23'57.8"W, 630 m/ Along La Escalera; 31.vii.2008/ leg. A. Short, M. García, L. Joly/ AS-08-058; rocky stream” (MIZA). ***Paratypes* (14 exs.): Venezuela: Bolívar State**: same data as holotype (14 exs., SEMC, including DNA voucher SLE1115).

##### Differential diagnosis.

See differential diagnosis for *Notionotusbicolor*. In addition, *N.insignitus* has a particular and unique color pattern in the elytra among the other congeners. The elytra are mostly dark brown, the posterior margins yellow and in the middle region has a characteristic yellow spot.

##### Description.

***Size and form***: Body length 1.6–1.8 mm. Body form elongate oval, convex in lateral view (Fig. 4D). ***Color and punctation***: Dorsally bicolor, head mostly brown, frons and middle region of the clypeus dark brown, lateral sides of the clypeus pale brown; pronotum yellow with two small black round spots along posterior margin; elytra dark brown, with a yellow spot on the anterior third of the elytra, lateral and posterior margins of the elytra yellow (Fig. 4D). Ventrally dark brown; maxillary palps, mouthparts, and antennae yellow (antennal club slightly darker). Pro and meso legs yellow, meta legs pale brown. Clypeus and labrum with dense, fine, and weakly impressed ground punctation (punctures separated by 2 × their width); pronotum and elytra ground punctation fine, weakly impressed and sparser than on head (punctures separated by 3 × their width). ***Head***: Clypeus and labrum shallowly emarginate anteromedially, lateral margins of the labrum bearing setae. ***Thorax***: Prosternum carinate medially, strongly raised, acute and pointing anteriorly. Elevation of mesoventrite with two transversal ridges, elevated medially, lateral sides concave; longitudinal ridge broad anteriorly and sharp posteriorly, the point where the three ridges merged wide and blunt (Fig. 10C); elevation flat in ventral view; mesoventrite with triangular shape in ventral view. Metaventrite convex in the median region, pubescent with narrow glabrous patch on the medial and posterolateral area; anterior margin extending to mesoventrite elevation. Metafemora with dense hydrofuge pubescence along basal three-quarters of the anterior margin and along basal one-quarter of the posterior margin, then anterior half of posterior margin with sparse setae. ***Abdomen***: Abdominal ventrites very densely pubescent. Aedeagus (Fig. 8B) with basal piece 0.6 × the length of a paramere. Base of the parameres narrower than the base of the median lobe; outer margin straight along basal two-thirds, then apically slightly convex, inner margins convex along basal two-thirds and then apically straight; basal two-thirds of the parameres broad then apical third narrower; apex of parameres rounded and pointing inwards. Median lobe shorter than the parameres, approximately triangular, with acute apex; gonopore triangular and situated at apex of median lobe.

##### Etymology.

The specific name comes from the Latin word *insignitus* meaning marked and refers to the distinctive yellow spot in the elytra of this species.

##### Distribution.

Known only from the type locality in southeastern Venezuela (Fig. 15).

##### Life history.

The only known series of this species was collected along the margin of a forested rocky stream (Fig. 12B).

#### ﻿ Notionotus juma sp. nov.

Taxon classificationAnimaliaColeopteraHydrophilidae

92D0546C-E5C7-510E-8DDE-602E9471C202

http://zoobank.org/DA88A467-D7C8-4AE1-A8E2-4033D8AF3364

**Figure 13.**
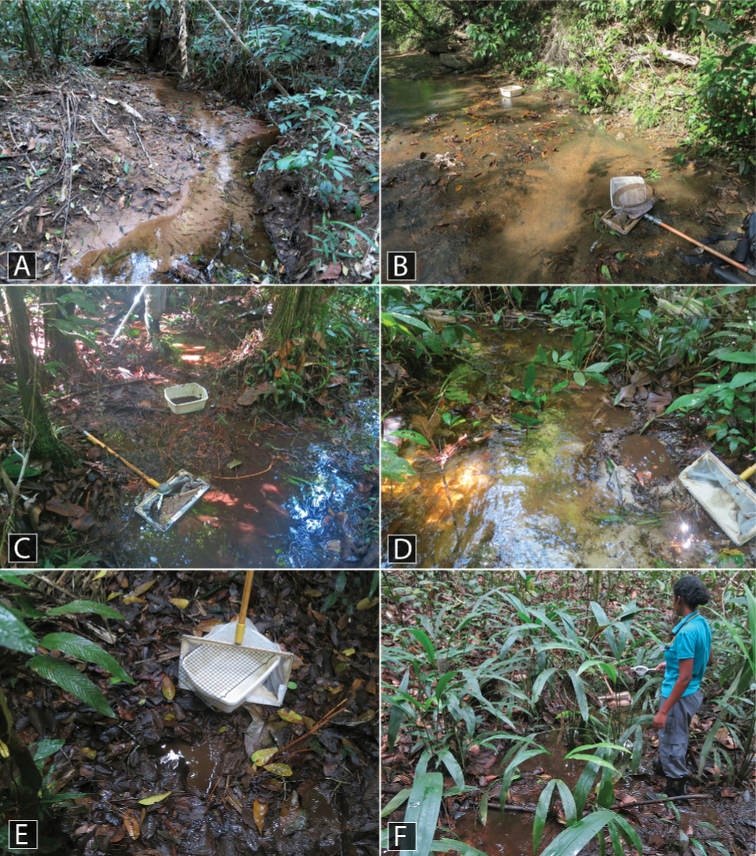
Habitat of *Notionotus* spp. **A** type locality and habitat of *N.giraldoi* (collecting event BR18-0710-02A) **B** type locality and habitat of *N.vatius* (collecting event BR18-0627-01A) **C** type locality and habitat of *N.brunbadius* (collecting event BR18-0609-03A) **D** type locality and habitat of *N.garciae* (collecting event BR18-0609-01A) **E** type locality and habitat of *N.juma* (collecting event BR18-0609-02B) **F** type locality and habitat of *N.retusus* (collecting event GY14-0311-02A).

Figs 4E , 8G , 13E , 15

##### Type material.

***Holotype* (male)**: “Brazil: Amazonas: Manaus/ -2.93079, -59.97514, 75 m/ Ducke Reserve, Igarape Barro/ Branco; Short & team; muddy/ pools in swampy area by stream/ 9.vi.2018; BR18-0609-02B” (INPA). ***Paratypes* (16 exs.): Brazil: Amazonas State**: Same data as holotype (13 exs., SEMC); same data except stream margins, 6.vi.2018, BR18-0606-02B (2 exs., SEMC, including DNA voucher SLE2100); Novo Airão Municipio, -2.68396, -60.93840, leg. Benetti, 9.vi.2017, densely vegetated margin of blackwater creek, BR17-0609-04A (1 ex., SEMC, DNA voucher SLE1269).

##### Differential diagnosis.

See differential diagnosis for *Notionotusgarciae*. In addition, the aedeagus of *N.juma* has an emargination in the apex of the medium lobe, being a particular feature among the species of the *lohezi* group.

##### Description.

***Size and form***: Body length 1.6–1.9 mm. Body form elongate oval, convex in lateral view (Fig. 4E). ***Color and punctation***: Dorsally bicolor, head pale brown, pronotum yellow with two small black round spots along posterior margin; elytra dark brown (Fig. 4E). Ventrally brown; maxillary palps, mouthparts, and antennae yellow (antennal club slightly darker). Pro and meso legs yellow, meta legs pale brown. Clypeus and labrum with dense, coarse, and moderately impressed ground punctation (punctures separated by 2 × their width); elytra and pronotum ground punctation coarse, moderately impressed and less dense than on head (punctures separated by 3 × their width). ***Head***: Clypeus and labrum shallowly emarginate anteromedially, lateral margins of the labrum bearing setae. ***Thorax***: Prosternum carinate medially, strongly raised, acute and pointing anteriorly. Elevation of mesoventrite with two transversal ridges, elevated medially, lateral sides concave; longitudinal ridge broad anteriorly and sharp posteriorly, the point where the three ridges merged wide and blunt (e.g., Fig. 10C, D); elevation flat in ventral view; mesoventrite with triangular shape in ventral view. Metaventrite convex in the median region, pubescent with narrow glabrous patch on the medial and posterolateral area; anterior margin extending to mesoventrite elevation. Metafemora densely covered with hydrofuge pubescence on basal three-quarters. ***Abdomen***: Abdominal ventrites very densely pubescent. Aedeagus (Fig. 8G) with basal piece nearly the same length as a paramere. Base of the parameres as wide as the base of the median lobe; outer margins convex, then curved inwards along apex, inner margins convex along basal two-thirds, and concave at apical third; apex of parameres rounded. Median lobe shorter than the parameres, wide at basal region, slightly narrowing in the midlength, apex wide and emarginate medially; gonopore with an oval shape and situated at apex of the median lobe.

##### Etymology.

This species is named after the Juma, an indigenous tribe located in the Açuã River, in the southern part of the state of Amazonas-Brazil.

##### Distribution.

Known only from the Ducke Reserve near Manaus, Brazil (Fig. 15).

##### Life history.

Specimens were collected in two habitats at the same forest reserve: in detrital pools in an area of shallowly flooded forest with detrital and mud substrate (Fig. 13E), and along the margins of a small stream.

#### ﻿ Notionotus lohezi

Taxon classificationAnimaliaColeopteraHydrophilidae

Queney, 2010

C7942A8C-21C0-5F87-921D-E469A5A301AA

Figs 8F , 15

Notionotus lohezi Queney, 2010: 135.

##### Type material examined.

***Paratype* (male)**: “♂ ”, “*Notionotuslohezi*/ n. sp. PARATYPE/ P. QUENEY descr. 2010”, “Guyane: Régina,/ Patawa, crique en/ forêt, 170 m,/ 13-IX-2009,/ leg. P. Queney” (SEMC). ***Paratypes* (5 exs.)**: same data as the paratype dissected (5 exs., SEMC).

##### Additional material examined

**(2 exs.). French Guiana**: Savane Roche Virginie, near RN 2, 4.1883, -52.13982, 64 m, Crique Chauve-souris, leg. Short, 10.iii.2020, solid granite substrate, detritus along margins on granite, FG20-0310-01A (2 exs., SEMC, including DNA vouchers SLE2337 and 2387).

##### Differential diagnosis.

See differential diagnosis for *Notionotusbicolor*.

##### Description.

***Size and form***: Body length 1.6–1.8 mm. Body form elongate oval, convex in lateral view (e.g., Fig. 4A). ***Color and punctation***: Dorsally bicolor, head bicolor, frons dark brown, clypeus yellow; pronotum yellow with two small black round spots along posterior margin; elytra dark brown, elytra margins paler (e.g., Fig. 4A). Ventrally dark brown; maxillary palps, mouthparts, and antennae yellow (antennal club slightly darker), legs brown. Clypeus and labrum with dense, fine, and weakly impressed ground punctation (punctures separated by 2 × their width); pronotum and elytra ground punctation fine, weakly impressed and sparser than on head (punctures separated by 3 × their width). ***Head***: Clypeus and labrum shallowly emarginate anteromedially, lateral margins of the labrum bearing setae. ***Thorax***: Prosternum carinate medially, strongly raised, acute and pointing anteriorly. Elevation of mesoventrite with two transversal ridges, elevated medially, lateral sides concave; longitudinal ridge broad anteriorly and sharp posteriorly, the point where the three ridges merged wide and blunt (e.g., Fig. 10C, D); elevation flat in ventral view; mesoventrite with triangular shape in ventral view. Metaventrite convex in the median region, pubescent with narrow glabrous patch on the medial and posterolateral area; anterior margin extending to mesoventrite elevation. Metafemora with dense hydrofuge pubescence along basal three-quarters of the anterior margin and along basal one-quarter of the posterior margin, then apical half of the posterior margin with sparse setae. ***Abdomen***: abdominal ventrites very densely pubescent. Aedeagus (Fig. 8F) with basal piece 0.8 × the length of the paramere. Base of the parameres slightly narrower than the base of the median lobe, inner margins straight, then convex reaching the apex, outer margins convex, and rounded apex pointing inwards. Median lobe shorter than the parameres, approximately rectangular with apex blunt; gonopore situated at the apex of the median lobe.

##### Distribution.

This species is only known from a few localities in French Guiana (Fig. 15).

##### Life history.

This species was collected in rocky streams with detritus along margins.

#### ﻿ Notionotus parvus sp. nov.

Taxon classificationAnimaliaColeopteraHydrophilidae

26E62B4C-A664-5394-A773-6CD2A7E2457E

http://zoobank.org/08140DE3-8B15-42F9-ADA9-3A1F952C1D3D

Figs 4F , 9E , 15

Notionotus sp. 2 in Short, 2013: 88.

##### Type material.

***Holotype* (male)**: “SURINAME: Sipaliwini District/ N 2.97731°, W 55.38500°, 200 m/ Camp 4 (low), Kasikasima; sandy/ stream on trail to METS camp/ 20.iii.2012; SR12-0320-02A/ leg. A. Short; 2012 CI-RAP Survey” (NZCS). ***Paratypes* (2 exs.): Suriname**: same data as holotype (2 exs., SEMC, including DNA voucher SLE2388).

##### Differential diagnosis.

*Notionotusparvus* can be recognized by the pale reddish yellow color in the head and pronotum and reddish brown in the elytra (Fig. 4F). Furthermore, the aedeagus is quite unique, parameres tubular in shape with lanceolate appendages in the outer margin which are shorter than the length of the parameres, and gonopore with crown-shape located in the apical region of the median lobe (Fig. 9E).

##### Description.

***Size and form***: Body length 1.7–1.9 mm. Body form elongate oval, convex in lateral view (Fig. 4F). ***Color and punctation***: Dorsally reddish brown, head and pronotum pale reddish yellow; pronotum with two small black round spots along posterior margin; elytra dark reddish brown (Fig. 4F). Ventrally reddish brown; maxillary palps and mouthparts light yellow reddish, and antennae yellow (antennal club slightly darker). Pro and meso legs yellow, meta legs pale reddish brown. Clypeus and labrum with dense, fine, and weakly impressed ground punctation (punctures separated by 2 × their width); pronotum and elytra ground punctation fine, weakly impressed and sparser than on head (punctures separated by 3 × their width). ***Head***: Clypeus and labrum shallowly emarginate anteromedially, lateral margins of the labrum bearing setae. ***Thorax***: Prosternum carinate medially, strongly raised, acute and pointing anteriorly. Elevation of mesoventrite with two transversal ridges, elevated medially, lateral sides concave; longitudinal ridge broad anteriorly and sharp posteriorly, the point where the three ridges merged wide and blunt (e.g., Fig. 10C, D); elevation slightly convex in ventral view; mesoventrite with triangular shape in ventral view. Metaventrite convex in the median region, pubescent with narrow glabrous patch on the medial and posterolateral area; anterior margin extending to mesoventrite elevation. Metafemora with dense hydrofuge pubescence along basal three-quarters of the anterior margin and sparse pubescence along the apical posterior margin. ***Abdomen***: Abdominal ventrites very densely pubescent. Aedeagus (Fig. 9E) with basal piece 0.5 × the length of a paramere. Base of the parameres narrower than the base of the median lobe; parameres tubular shape, outer margins sinuate and covered by lanceolate appendages that cover three-quarters of the margin; inner margins sinuate, apex of the parameres rounded. Median lobe shorter than the parameres, wide at basal region, narrowing apically, apex rounded and wide; gonopore crown-shaped and situated at apex of median lobe.

**Figure 14.**
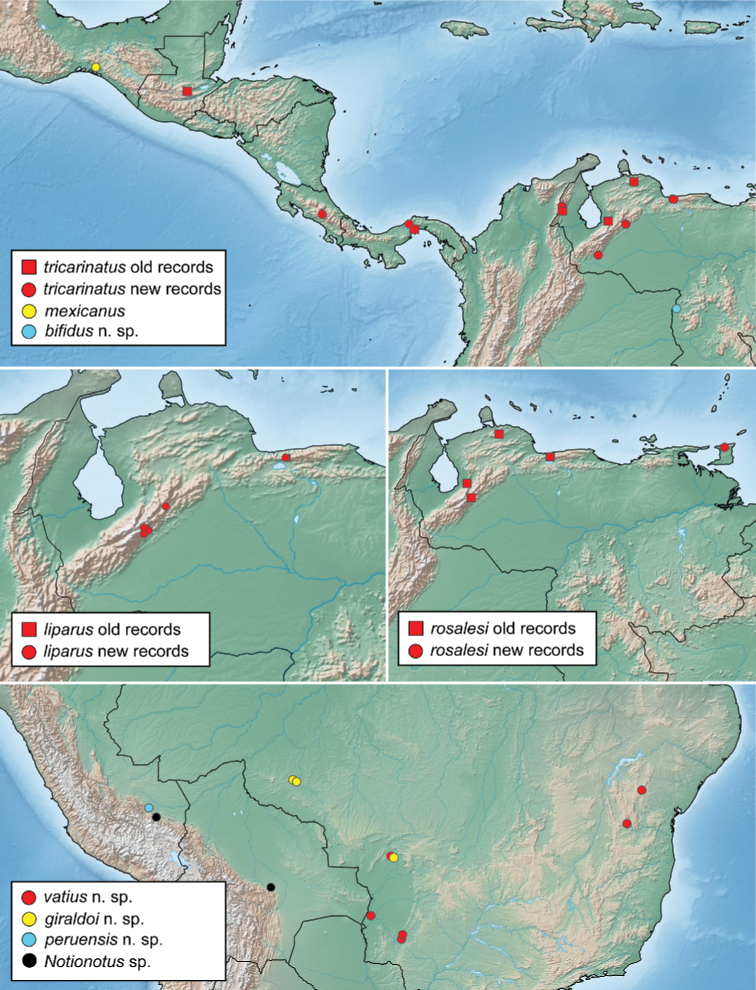
Distribution map of *Notionotus* spp.

##### Etymology.

The species name is derived from the Latin word *parvus* meaning little or small in reference to the small aedeagus size of this species.

##### Distribution.

**Figure 15.**
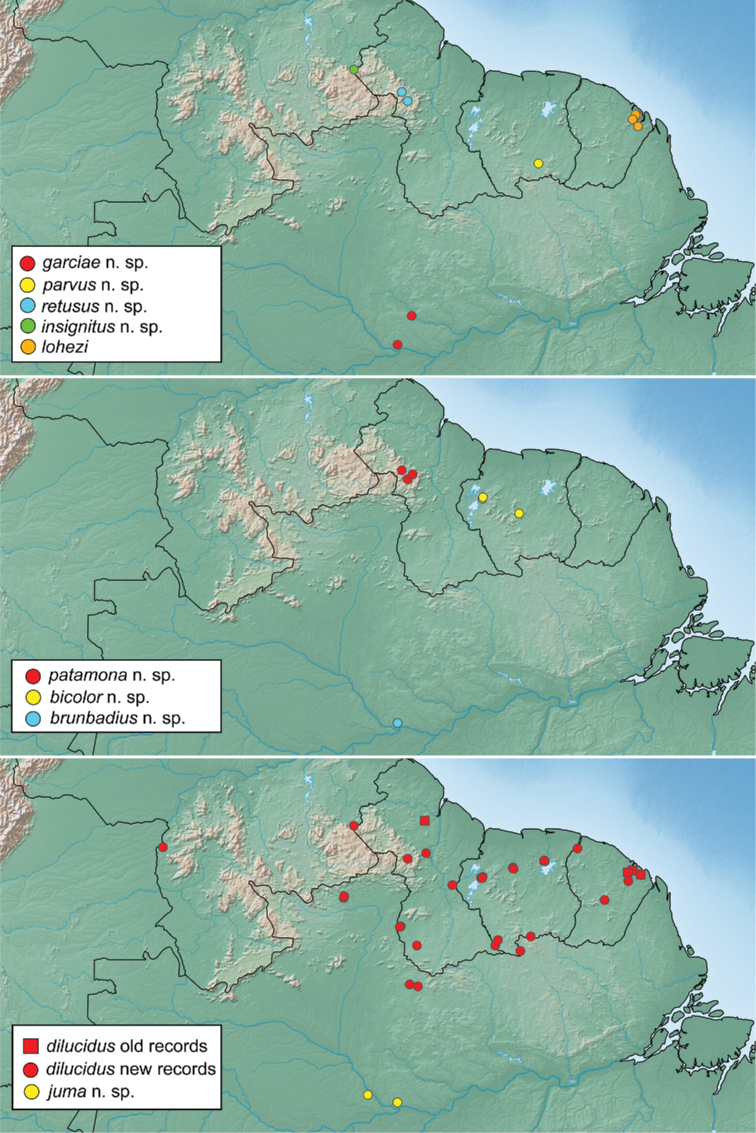
Distribution map of *Notionotus* spp.

Only known from the type locality in southern Suriname (Fig. 15).

##### Life history.

This species was collected along the margins of a small, sandy-bottomed stream.

#### ﻿ Notionotus patamona sp. nov.

Taxon classificationAnimaliaColeopteraHydrophilidae

468569EE-C5E1-58F9-9A09-D8B66D975E7C

http://zoobank.org/B8AC3A70-8620-44E5-8665-709DCF4307AA

Figs 4B , 8D , 12C , 15

##### Type material.

***Holotype* (male)**: “GUYANA: Region XIII [!sic: Region 8]/ 5°18.264'N, 59°50.257'W; 687 m/ Ayanganna Airstrip; trail from air-/ strip to Ayanganna; seepage area; over rocks in forest flowing into/ stream; leg. A. Short; 18.iii.2014/ GY14-0318-01C” (CBDG). ***Paratype* (12 exs.): Guyana: Region 8**: Same data as holotype (8 exs., SEMC); Upper Potaro Camp I (ca. 7 km NW Chenapau), Potaro margin trail, 5°0.660'N, 59°38.283'W, 484 m, 11.iii.2014, leg. Short, Baca, Salisbury and La Cruz, wet detritus in sandy area, GY14-0311-04A (1 ex., SEMC); top of falls on Potaro River, 5°0.730'N, 59°38.965'W, 585 m, 12.iii.2014, leg. Short, Salisbury and La Cruz, seeps with roots and algae, GY14-0312-01B (1 ex., SEMC); stream near camp, 5°0.673'N, 59°38.358'W, 500 m, 14.iii.2014, leg. Short, Salisbury and La Cruz, gravel/sandy stream w/ detritus, GY14-0314-01A (1 ex., SEMC); Kaieteur Natural Park, trail by guest house, 5°10.514'N, 59°28.970'W, 440 m, 21.iii.2014, leg. Short, Salisbury and La Cruz, forest pools, GY14-0321-01B (1 ex., SEMC).

##### Differential diagnosis.

See differential diagnosis for *Notionotusbicolor*.

##### Description.

***Size and form***: Body length 1.6–1.8 mm. Body form elongate oval, convex in lateral view (Fig. 4B). ***Color and punctation***: Dorsally bicolor, head brown, frons dark brown, clypeus pale brown; pronotum yellow with two small black round spots along posterior margin; elytra dark brown, elytra margins paler (Fig. 4B). Ventrally brown; maxillary palps, mouthparts, antennae, and legs yellow. Clypeus and labrum with dense, fine, and weakly impressed ground punctation (punctures separated by 2 × their width); pronotum and elytra ground punctation fine, weakly impressed and sparser than on head (punctures separated by 3 × their width). ***Head***: Clypeus and labrum shallowly emarginate anteromedially, lateral margins of the labrum bearing setae. ***Thorax***: Prosternum carinate medially, strongly raised, acute and pointing anteriorly. Elevation of mesoventrite with two transversal ridges, elevated medially, lateral sides concave; longitudinal ridge broad anteriorly and sharp posteriorly, the point where the three ridges merged wide and blunt (e.g., Fig. 10C, D); elevation flat in ventral view; mesoventrite with triangular shape in ventral view. Metaventrite convex in the median region, pubescent with narrow glabrous patch on the medial and posterolateral area; anterior margin extending to mesoventrite elevation. Metafemora with dense hydrofuge pubescence along basal three-quarters of the anterior margin and along basal one-quarter of the posterior margin, then apical half of the posterior margin with sparse setae. ***Abdomen***: Abdominal ventrites very densely pubescent. Aedeagus (Fig. 4B) with basal piece 0.7 × the length of a paramere. Base of the parameres narrower than the base of the median lobe; outer margins straight along basal two-thirds, then apically slightly convex, inner margins straight along basal two-thirds and then convex and tapering apically; apex of parameres rounded and pointing inwards. Median lobe shorter than the parameres, approximately triangular, gradually narrowing from the base, broad and rounded apex; gonopore oval-shaped and situated at apex of median lobe.

##### Etymology.

This species is named after the Patamona, an indigenous tribe located in the mountainous region from which this species is known.

##### Distribution.

Known from several closely situated localities in western Guyana (Fig. 15).

##### Life history.

This species was collected at several a variety of stream-associated habitats, including along the margins of detritus and sandy-based streams, as well as in rock seepage habitats adjacent to streams (Fig. 12C).

#### ﻿ Notionotus retusus sp. nov.

Taxon classificationAnimaliaColeopteraHydrophilidae

33F55D59-C29C-5283-8EC6-CDB4E3BD0670

http://zoobank.org/366A532F-DAAF-4521-9E0B-CD6AACA17266

Figs 8E , 13F , 15

##### Type material.

***Holotype* (male)**: “GUYANA: Region XIII [!sic: Region 8]/ 5°0.730'N, 59°38.965'W, 585 m/ Upper Potaro Camp I (c. 7 km/ NW Chenapau), Ridge Trial/ leg. Short, Baca and Salisbury/ 11.iii.2014; GY14-0311-02A”, “DNA VOUCHER/ Extraction #/ SLE-2110” (CBDG). ***Paratype* (1 ex.): Guyana: Region 8**: Ayanganna Airstrip, 5°18.264'N, 59°50.257'W; 687 m, trail from airstrip to Ayanganna, leg. A. Short, 18.iii.2014, seepage area over rocks in forest flowing into stream, GY14-0318-01C (1 ex., SEMC, DNA voucher 2372)

##### Differential diagnosis.

The external characters of *Notionotusretusus* and *N.lohezi* are quite similar. The only way they can be separated is by the features of the aedeagus. In this species, the apex of the parameres is wide and rounded (acute in *N.lohezi*, Fig. 8F), the median lobe is much longer than in *N.lohezi* and the gonopore is elongated and oval (nearly rectangular in *N.lohezi*).

##### Description.

***Size and form***: Body length 1.8 mm. Body form elongate oval, moderately convex in lateral view. ***Color and punctation***: Dorsally bicolor, head mostly brown, frons brown, clypeus pale brown; pronotum yellow with two small black round spots along posterior margin; elytra dark brown. Ventrally brown; maxillary palps, mouthparts, antennae (antennal club slightly darker), legs brown. Clypeus and labrum with dense, fine, and weakly impressed ground punctation (punctures separated by 2 × their width); pronotum and elytra ground punctation fine, weakly impressed, and sparser than on head (punctures separated by 3 × their width). ***Head***: Clypeus and labrum shallowly emarginate anteromedially, lateral margins of the labrum bearing setae. ***Thorax***: Prosternum carinate medially, strongly raised, acute and pointing anteriorly. Elevation of mesoventrite with two transversal ridges, elevated medially, lateral sides concave; longitudinal ridge broad anteriorly and sharp posteriorly, the point where the three ridges merged wide and blunt (e.g., Fig. 10C, D); elevation flat in ventral view; mesoventrite with triangular shape in ventral view. Metaventrite convex in the median region, pubescent with narrow glabrous patch on the medial and posterolateral area; anterior margin extending to mesoventrite elevation. Metafemora with dense hydrofuge pubescence along basal three-quarters of the anterior margin and along basal one-quarter of posterior margin, then apical half of the posterior margin with sparse setae. ***Abdomen***: Abdominal ventrites very densely pubescent. Aedeagus (Fig. 8E) with basal piece 0.7 × the length of a paramere. Base of the parameres nearly the same as the base of the median lobe; outer margins convex, inner margins nearly straight; apex of parameres broad, rounded and slightly pointing inwards. Median lobe shorter than the parameres, wide at basal region, narrowing in basal third, margins straight and apex rounded and nearly notched; gonopore with an oval shape and covering approximately two-thirds of the length of the median lobe.

##### Etymology.

The specific name comes from the Latin word *retusus* meaning rounded and notched, after the form of the apex of the median lobe of the aedeagus.

##### Distribution.

This species is only known from the type locality in western Guyana (Fig. 15).

##### Life history.

This species was collected in detrital-filled pools in a shallow ravine with dense forest cover (Fig. 13F). Although the pools might not be considered a stream, the pools were part of a drainage network.

###### *Notionotusrosalesi* species group

**Diagnosis.** The species of this group can be recognized by the unique dorsal coloration, being almost yellow with a wide brown band in the third anterior of the elytra (Fig. 2G), the elevation of the mesoventrite with one transversal and longitudinal ridge (e.g., Fig. 10B), the shape of the genitalia is quite distinct, being the only one within all *Notionotus* species of the Neotropical region with the apical third of the parameres membranous (Fig. 9A–C).

#### ﻿ Notionotus rosalesi

Taxon classificationAnimaliaColeopteraHydrophilidae

Spangler, 1972

FC97C583-DD21-5ACB-89CC-E8A5BE6E3D01

Figs 2G–I , 9A–C , 14

Notionotus rosalesi Spangler, 1972: 141

##### Type material examined.

**Holotype (male)**: “VENEZUELA/Arag., 10 Km S./Rancho Grande/II-14-1969/P.&P. Spangler”, “TypeNo/71950/U S N M”, “HOLOTYPE/ Notionotus/rosalesi/P.J.Spangler” (USNM).

##### Additional material examined.

**Trinidad: Guanapo State**: 4.1 km up Guanapo Valley, trib of Guanapo River, 460 ft, 11-VII-2005 (1 ex., SEMC); Verdant vale, Arima River, 10°42'N, 61°18'W, 570 ft, 9-VII-2005 (1 ex., SEMC). **Venezuela: Aragua**: Rancho Grande Biol. Stn. 1150 m, 10°21'N, 67°41'W, 25–28 II 1995, S. Marshall, yellow pan trap (1 ex., SEMC).

##### Differential diagnosis.

*Notionotusrosalesi* can be distinguished by the wide brown band in the anterior third of the elytra, as well as, the unique shape of the genitalia, having many accessories at the base of the parameres, the apex of the parameres membranous and lanceolate.

##### Description.

***Size and form***: Body length 1.8–1.9 mm. Body form elongate oval, moderately convex in lateral view (Fig. 2G). ***Color and punctation***: Dorsally bicolor, head mostly brown, frons brown, medially region of the clypeus pale brown with lateral margins yellow; pronotum yellow with two small black round spots along posterior margin; elytra yellow except by a brown wideband on the anterior third of the elytra (Fig. 2G). Ventrally brown; maxillary palps, mouthparts, antennae, and legs yellow (antennal club slightly darker) (Fig. 2H). Clypeus and labrum with dense, fine, and weakly impressed ground punctation (punctures separated by 5 × their width); pronotum and elytra ground punctation fine, weakly impressed and sparser than on head (punctures separated by 3 × their width). ***Head***: Clypeus and labrum shallowly emarginate anteromedially, lateral margins of the labrum bearing setae. ***Thorax***: Prosternum carinate medially, strongly raised, pointing anteriorly and acute. Elevation of mesoventrite with one transversal ridge, elevated medially, lateral sides concave; longitudinal ridge narrowed anteriorly and broadening posteriorly, the point where the two ridges merged acute (e.g., Fig. 10B); elevation concave in lateral view; mesoventrite with triangular shape in ventral view. Metaventrite convex in the median region, pubescent with narrow glabrous patch on the medial and posterolateral area; anterior margin extending to mesoventrite elevation. Metafemora with dense hydrofuge pubescence along basal three-quarters of the anterior margin and along basal half of the posterior margin. ***Abdomen***: Abdominal ventrites very densely pubescent. Aedeagus (Fig. 9A–C) basal piece 0.4 × the length of a paramere; broad parameres, base of the parameres much wider than the base of the median lobe, base of the parameres with two accessories with ovate shape, outer margins of parameres strongly sinuate, inner margins slightly sinuate, with membranous acuminate apex, bending outwards; median lobe shorter than the parameres, approximately triangular, with apex rounded.

##### Distribution.

Originally described from the Venezuelan states of Aragua and Barinas (Spangler 1972), it was later reported from the states of Trujillo and Falcón (García 2000). Here we report it for the first time from Trinidad (Fig. 14).

##### Life history.

Although specific habitat information is limited, all specimens were collected in association with streams. Spangler (1972) characterized this species as a hygropetric specialist, although not all specimens known at that time were from seepages (the others were from a stream pool that “was in the bedrock and the bottom was covered with rotting leaves”).

##### Remarks.

The genitalia of the holotype appears to have some modest fungal growth on the median lobe (Fig. 9B), the circular “halo” at the tip of the median lobe appears to be unnatural and is not part of the original structure.

###### *Notionotusperuensis* species group

**Diagnosis.** The species of *peruensis* group can be diagnosed by the dorsal coloration completely yellow, the elevation of the mesoventrite with one transverse and one longitudinal (e.g., Fig. 10B), and by the shape of the genitalia (Fig. 9F).

#### ﻿ Notionotus peruensis sp. nov.

Taxon classificationAnimaliaColeopteraHydrophilidae

404A2042-5109-524C-B4D7-B2D2F84D3860

http://zoobank.org/4E05420D-577E-4B12-8B2B-783B0C0BB101

Figs 4K , 9F , 14

##### Type material examined.

***Holotype* (male)**: “PERU: Dept. Madre de/ Dios: Pantiacolla Lodge,/ Alto Madre de Dios R./ 12°39.3'S, 71°13.9'W 420 m/ 14–19-XI-2007 D. Brzoska/ ex. flight intercept trap/ PER1B07 004” (SEMC).

##### Differential diagnosis.

*Notionotusperuensis* can be distinguished by the particular shape of the aedeagus, being nearly rectangular in the basal half and abruptly narrow in the apical half (Fig. 9F).

##### Description.

***Size and form***: Body length 1.6 mm. Body form elongate oval, convex in lateral view (Fig. 4K). ***Color and punctation***: Dorsally yellow, head mostly yellow and frons pale brown; pronotum with two small black round spots along posterior margin (Fig. 4K). Ventrally brown; maxillary palps, mouthparts, antennae (antennal club slightly darker) and legs yellow. Clypeus and labrum with dense, fine, and weakly impressed ground punctation (punctures separated by 2 × their width); pronotum and elytra ground punctation fine, weakly impressed and sparser than on head (punctures separated by 3 × their width). ***Head***: Clypeus and labrum shallowly emarginate anteromedially, lateral margins of the labrum bearing setae. ***Thorax***: Prosternum carinate medially, strongly raised, pointing anteriorly and acute. Elevation of mesoventrite with one transversal ridge, elevated medially, lateral sides concave; longitudinal ridge sharp, the point where the two ridges merged acute (e.g., Fig. 10B); elevation flat in lateral view; mesoventrite with triangular shape in ventral view. Metaventrite convex in the median region, pubescent with narrow glabrous patch on the medial and posterolateral area, medial region patch drop-shaped; anterior margin extending to mesoventrite elevation. Metafemora densely covered with hydrofuge pubescence on basal three-quarters. ***Abdomen***: Abdominal ventrites very densely pubescent. Aedeagus (Fig. 9F) with basal piece 0.7 × the length of a paramere. Base of the parameres wider than the base of the median lobe; outer margins straight along basal two-thirds, then apically sinuate, inner margins straight along basal two-thirds and then convex and tapering apically; apex of parameres rounded and pointing outwards. Median lobe much shorter than the parameres, basal half rectangular, apical half narrowing abruptly, apex rounded.

##### Etymology.

The species is named after Peru, the country where it was collected, as well as for being the first species described for the genus in this country.

##### Distribution.

Known only from the type locality in Peru (Fig. 14).

##### Life history.

The single specimen was collected at a flight intercept trap; nothing is known about its habitat.

#### ﻿ Notionotus spp.

Taxon classificationAnimaliaColeopteraHydrophilidae

F684E1D5-2402-5BCE-A17A-D5836C0061D6

Fig. 14

##### Material examined

**(2 exs.). Bolivia: Santa Cruz**: Amboro National Park, Guarda Parque Mataracu, 21–27.xi.2004, malaise trap, leg. Robertson, García, & Vidaurre (1 female, UMSP). **Peru: Cusco**: Quispicanchi Province, streams 1 km N Quince Mil, 13°13.335'S, 70°46.035'W, 730 m, 9.i.2020, leg. Baca., Slow rivulets & pools w/ saturated detritus next to stream, PE20-0109-02A (1 female, SEMC, DNA voucher SLE2140).

##### Remarks.

We examined two single female specimens from unique localities that we refrain from identifying or describing, due to the lack of male genitalia for comparison. The specimen from Peru likely represents an undescribed species, as suggested by its distant position in our DNA tree (Fig. 1). The single female from Bolivia is the first and only known record of the genus from that country.

### ﻿Key to the species groups of *Notionotus* of the Neotropical Region

Although it is fairly straightforward to key specimens (especially males) to species group, identification to species within each group almost always requires examination of the aedeagus. For this reason, as well as the fact that there are no doubt many yet-to-be-described species, particularly in the southern Amazon region, we do not include a species key here.

**Table:** 

1	Elevation of the mesoventrite composed by two ridges (one transverse and one longitudinal) (e.g., Fig. 10A, B)	**2**
–	Elevation of the mesoventrite composed by three ridges (two transverse and one longitudinal) (e.g., Fig. 10C, D)	***lohezi* species group**
2	Length of the parameres nearly the same as the length of the basal piece. Length of the median lobe almost the same as the parameres (e.g., *N.liparus*, Fig. 7A)	***liparus* species group**
–	Length of the parameres longer than the basal piece. Length of the median lobe almost the same as the parameres	**3**
3	Outer margin of the parameres convex and tapering reaching the apex, rounded apex. Median lobe rectangular along basal half then tapering abruptly (e.g., *N.peruensis* sp. nov., Fig. 9F)	***peruensis* species group**
–	Outer margin of the parameres sinuate, apex of the parameres lanceolate. Median lobe wide and approximately triangular (e.g., *N.rosalesi*, Fig. 9F)	***rosalesi* species group**

## ﻿Discussion

Since *Notionotus* was first described as a hygropetric genus from the Andean Region of Venezuela fifty years ago (Spangler 1972), our knowledge of the genus has substantially expanded. Within the Neotropical region, this present work expands the distribution throughout much of tropical South America, including the first records from Peru, Bolivia, and Brazil. We also find that while the originally described species do appear to prefer hygropetric habitats (especially *N.liparus*), most species are not found in seepages but prefer leaf packs and margins of forested streams. The vast majority of specimens have been collected at sites under 1000 meters in elevation, though *N.liparus* has been found as high as 1770 m. Although the highest density of known specimen records remains in the northern quarter of South America, particularly Venezuela and the Guiana Shield region, this is almost certainly an artifact of collecting effort, as aquatic beetles are in general less well known in the central and southern Amazon region. While many new species undoubtedly remain to be described, we also found that some species have quite expansive ranges (e.g., *N.dilucidus*, *N.tricarinatus*, *N.vatius*) and care should be taken to put even seemingly geographically distant new collections in context with existing species. The integration of DNA sequence data proved invaluable in helping to establish species limits in these widespread taxa.

## Supplementary Material

XML Treatment for Notionotus

XML Treatment for Notionotus dilucidus

XML Treatment for Notionotus shorti

XML Treatment for Notionotus giraldoi

XML Treatment for Notionotus liparus

XML Treatment for Notionotus mexicanus

XML Treatment for Notionotus tricarinatus

XML Treatment for Notionotus edibethae

XML Treatment for Notionotus nucleus

XML Treatment for Notionotus perijanus

XML Treatment for Notionotus vatius

XML Treatment for Notionotus bicolor

XML Treatment for Notionotus bifidus

XML Treatment for Notionotus brunbadius

XML Treatment for Notionotus garciae

XML Treatment for Notionotus insignitus

XML Treatment for Notionotus juma

XML Treatment for Notionotus lohezi

XML Treatment for Notionotus parvus

XML Treatment for Notionotus patamona

XML Treatment for Notionotus retusus

XML Treatment for Notionotus rosalesi

XML Treatment for Notionotus peruensis

XML Treatment for Notionotus
